# Structure–function characterization of the mono- and diheme forms of MhuD, a noncanonical heme oxygenase from *Mycobacterium tuberculosis*

**DOI:** 10.1016/j.jbc.2021.101475

**Published:** 2021-12-06

**Authors:** Samuel N. Snyder, Piotr J. Mak

**Affiliations:** Department of Chemistry, Saint Louis University, Saint Louis, Missouri, USA

**Keywords:** MhuD, Mycobacterium tuberculosis, heme oxygenase, Raman spectroscopy, diheme, protein chemistry, enzyme catalysis, 5cHS, 5-coordinated high spin, 6cHS, 6-coordinated high spin, 6cLS, 6-coordinated low spin, ESI-MS, electrospray ionization mass spectrometry, HF, high frequency, His, histidine, HO, heme oxygenase, hHO-1, human heme oxygenase-1, LF, low frequency, Mtb, *Mycobacterium tuberculosis*, oop, out-of-plane, POR, NADPH-cytochrome P450 oxidoreductase, PPIX, protoporphyrin IX, rR, resonance Raman, UV-vis, ultraviolet-visible

## Abstract

MhuD is a noncanonical heme oxygenase (HO) from *Mycobacterium tuberculosis* (Mtb) that catalyzes unique heme degradation chemistry distinct from canonical HOs, generating mycobilin products without releasing carbon monoxide. Its crucial role in the Mtb heme uptake pathway has identified MhuD as an auspicious drug target. MhuD is capable of binding either one or two hemes within a single active site, but only the monoheme form was previously reported to be enzymatically active. Here we employed resonance Raman (rR) spectroscopy to examine several factors proposed to impact the reactivity of mono- and diheme MhuD, including heme ruffling, heme pocket hydrophobicity, and amino acid–heme interactions. We determined that the distal heme in the diheme MhuD active site has negligible effects on both the planarity of the His-coordinated heme macrocycle and the strength of the Fe-N_His_ linkage relative to the monoheme form. Our rR studies using isotopically labeled hemes unveiled unexpected biomolecular dynamics for the process of heme binding that converts MhuD from mono- to diheme form, where the second incoming heme replaces the first as the His75-coordinated heme. Ferrous CO-ligated diheme MhuD was found to exhibit multiple Fe-C-O conformers, one of which contains catalytically predisposed H-bonding interactions with the distal Asn7 residue identical to those in the monoheme form, implying that it is also enzymatically active. This was substantiated by activity assays and MS product analysis that confirmed the diheme form also degrades heme to mycobilins, redefining MhuD’s functional paradigm and further expanding our understanding of its role in Mtb physiology.

Degradation of heme is a critically important physiological process in many forms of life catalyzed by heme oxygenases (HOs). Canonical HOs degrade heme to biliverdin, releasing carbon monoxide and free ferrous iron (Fig. 1) (1, 2), and this activity was considered a paradigm for all heme degradation until the recent discovery of some bacterial HOs that catabolize heme to different products. MhuD from *Mycobacterium tuberculosis* (Mtb) degrades heme to mycobilins, which retain the α-meso carbon as an aldehyde at the ring cleavage site (3). IsdG and IsdI from *Staphylococcus aureus* are other examples of noncanonical HOs that degrade heme to staphylobilins, releasing Fe(II) and formaldehyde instead of CO (4, 5). The distinct products of canonical and noncanonical HOs suggest different enzymatic mechanisms, presumably involving different reactive intermediates. In pathogens, the main function of HOs is to harvest the iron needed to survive and sustain infections from host heme molecules (6). The essential role of MhuD in the Mtb heme uptake pathway has identified it as an auspicious target for development of new antitubercular drugs and treatment strategies (7, 8).

**Figure 1:**
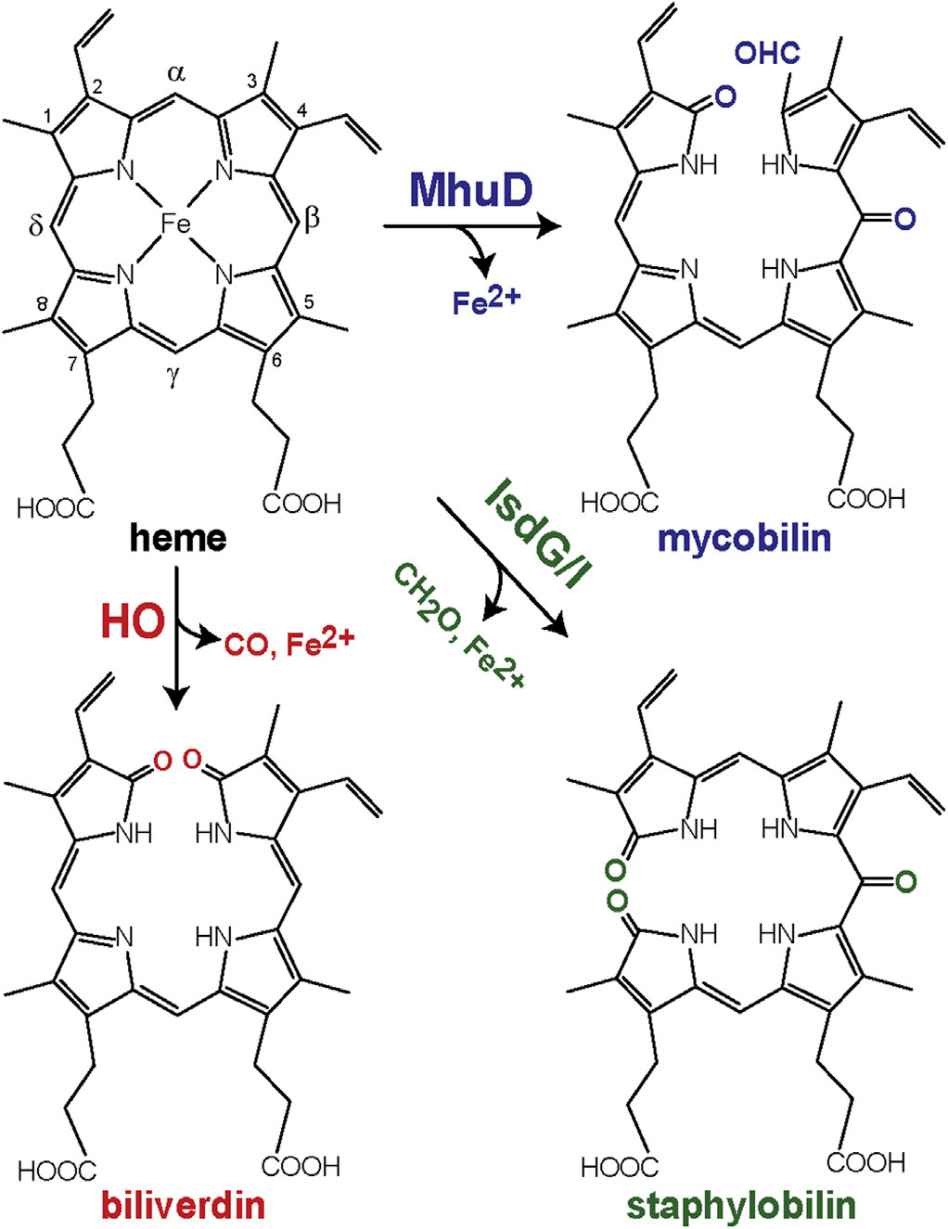
**Structures of heme and its degradation products achieved through different heme oxygenase pathways.** The canonical HO product, α-biliverdin is labeled in *red*. The noncanonical HO products mycobilin (mycobilin-a) from MhuD and staphylobilin (5-oxo-δ-bilirubin) from IsdG/I pathways are labeled in *blue* and *green*, respectively.

MhuD and IsdG/I proteins form homodimers of subunits with ferredoxin-like α/β-sandwich folds and mostly hydrophobic distal active sites containing only one polar amino acid residue (asparagine) and no ordered water molecules (9, 10, 11, 12). Conversely, canonical HOs have an overall α-helical fold and a highly polar distal heme pocket containing two ordered water molecules that are essential for its enzymatic activity (13, 14). Interestingly, MhuD is capable of binding either one or two hemes within the same active site, a feature that is unique among all known HOs (Fig. 2). The heme in the monoheme form of MhuD is coordinated to the proximal His-75 residue. In the diheme form, the additional heme was proposed to be in the distal active site stacked planar upon the His-ligated heme and coordinated indirectly with Asn-7 *via* a chloride ion (10). Asn-7 was suggested to be key to the enzymatic activity of MhuD by providing H-bonding interactions to stabilize and regiospecifically orient reactive intermediates in the monoheme form (3), an interaction that structural data suggested to be blocked by the distal heme in diheme MhuD (10). Additionally, the heme macrocycle in monoheme MhuD was reported to exhibit unusually extensive ruffling similar to IsdG/I proteins, as opposed to more planar heme structures in diheme MhuD and canonical HOs. This distortion was implied to contribute to different heme degradation mechanisms of noncanonical HOs (11, 15, 16).

**Figure 2:**
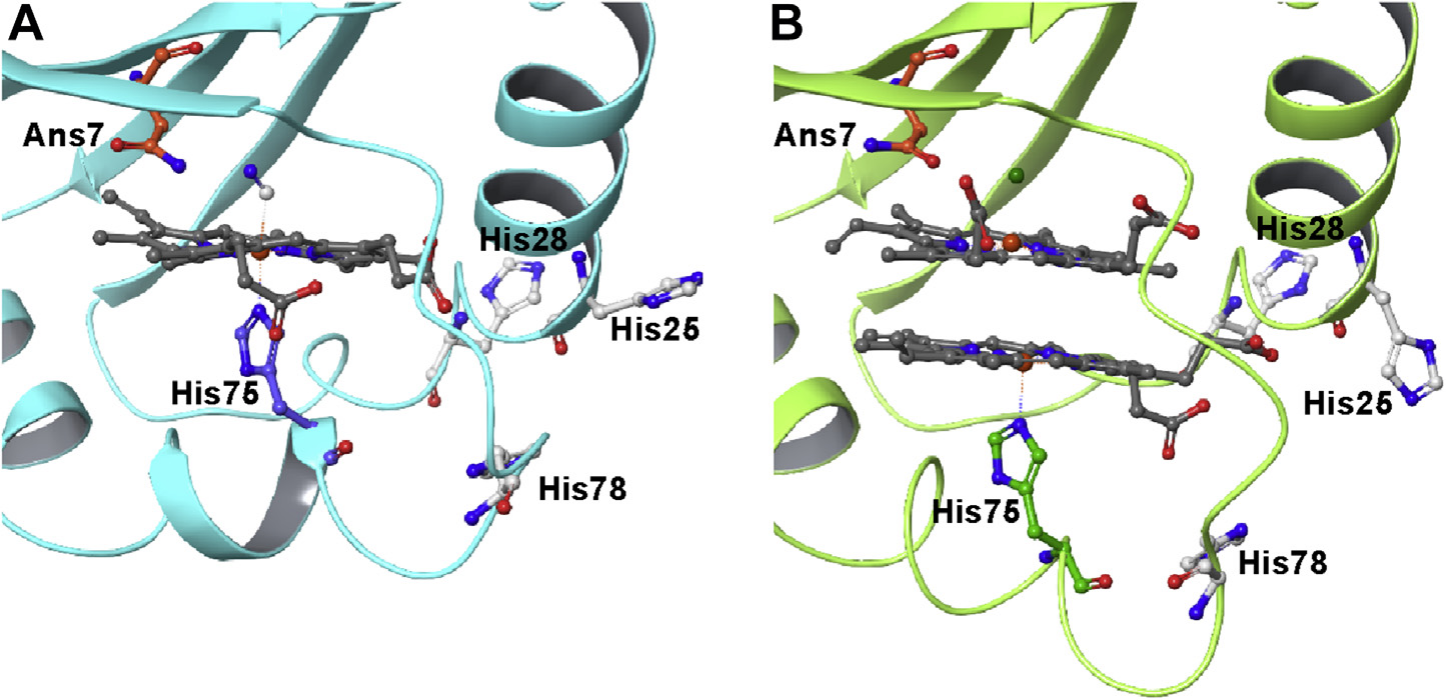
**X-ray crystal structures showing the active sites of monoheme and diheme forms of MhuD.** Panel *A* shows the 6-coordinated low spin ferric-CN ligated monoheme MhuD structure (PDB ID 4NL5) with the protein secondary structure displayed in *light blue* (11). Panel *B* shows ferric diheme MhuD with both hemes in the 5-coordinated high spin state (PDB ID 3HX9) with the protein secondary structure displayed in *light green* (10). In both structures, the side chain functional groups of the following amino acids are displayed: Asn-7, His-25, His-28, His-75, and His-78. The proximal heme in both forms is shown coordinated to His-75, despite the presence of several other histidine residues surrounding the active site. The α2 helix is kinked in monoheme but is extended in the diheme form to expand the volume of the active site to accommodate an additional heme. The Asn-7 residue is shown within range of electrostatic interactions with the exogenous cyanide ligand in monoheme, and the Cl^−^ ion (*green sphere*) coordinated axially to the distal heme in the diheme form.

It was shown that MhuD degrades heme by unprecedented sequential mono- and dioxygenation reactions (17). The initial studies showed that the monoheme form was enzymatically active, while diheme was inactive (10). This brought into question the functional role of the second heme in the catalysis of MhuD. It was proposed that MhuD has much higher affinity for the first heme than the second, implying that monoheme functions as a heme degrader in low intracellular heme conditions and when heme concentrations rise, MhuD binds the second heme, and its diheme form acts as a heme storage or regulatory protein (18). However, more recent studies showed that MhuD did not evolve to preferentially bind one heme molecule and that the diheme form is comparably favored, implicating a more complex functional role of diheme MhuD in Mtb physiology (19).

Resonance Raman (rR) spectroscopy is an ideal technique for studying the active site environment of heme proteins, providing a wide range of structural information regarding oxidation, spin, and coordination states of heme iron, geometries of heme substituent groups and their H-bonding interactions, deformations of the heme plane, and the disposition of endogenous and exogenous heme axial ligands (20, 21, 22, 23, 24, 25). As such, rR studies are particularly well suited for revealing structural differences between mono- and diheme MhuD as they were proposed to exhibit differences in heme planarity (10, 11). rR measurements of ferrous His-coordinated heme proteins reveal the strength of the Fe-proximal ligand bond (23). Additionally, rR studies of ferrous carbonmonoxy adducts of heme proteins provide an effective probe of the polarity and crowding of the distal active site environment (26, 27), making the CO adduct ideal for monitoring the MhuD heme pocket in the presence or absence of an additional heme.

## Results

### Preparation of MhuD protein

The presence of a terminal His-tag on MhuD was previously shown to complicate the determination of heme-binding affinity (18). To obtain the most accurate model of MhuD protein without any additional amino acids to its native primary structure from affinity tags or protease cleavage sites, we employed a fusion of MhuD at its C-terminus to a cleavable intein, DNA gyrase subunit A from *Mycobacterium xenopi* (Mxe GyrA), which contains a chitin-binding domain that allows affinity purification prior to its removal in its entirety from MhuD. This expression and purification process produced relatively high yields of apo-MhuD protein, *e.g.*, ∼20 mg per liter of cell culture and >99% purity determined by SDS-PAGE (Fig. S1) and Image Lab software (Bio-Rad).

Reconstituting apo-MhuD protein with heme is also not a trivial endeavor. Since MhuD can bind two hemes in the same active site, special measures must be taken to ensure reliable and consistent heme:protein stoichiometry during reconstitution of monoheme samples to prevent formation of diheme, which was shown to occur even when incubated with heme in a 1:1 molar ratio (19). Therefore, MhuD samples in this study were reconstituted using a CN-CO replacement method that has provided an apparent solution to this challenging problem. The effectiveness of this method in producing pure ferric monoheme MhuD without contamination by residual diheme populations is evidenced by an almost 1:1 molar ratio of heme to protein despite being incubated with approximately fourfold excess of hemin-dicyanide. This new technique used to generate mono- and diheme samples with ∼1:1 and ∼2:1 heme:protein stoichiometric ratios, respectively, was crucial to accurately characterize the structural differences between the two forms of MhuD. Additional results and detailed discussion for the preparation of MhuD protein samples are provided in the Supporting information.

### Ferric state

**Figure 3:**
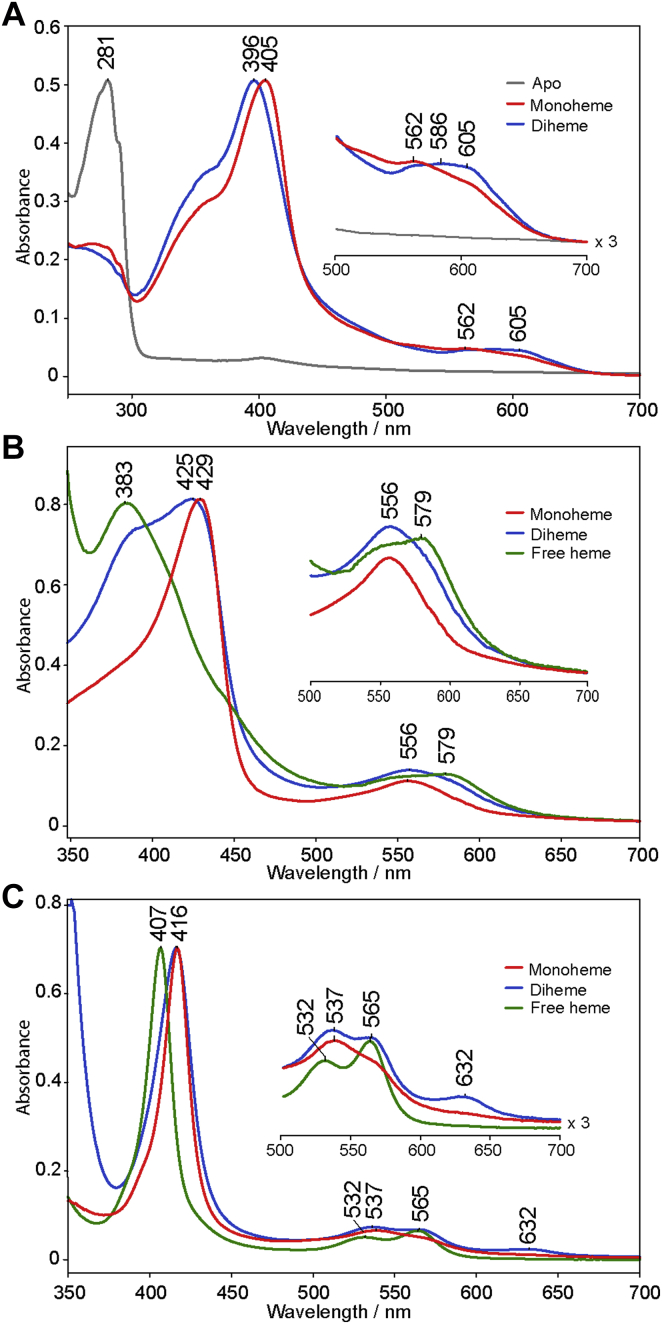
**UV-vis spectra of mono- and diheme MhuD and free heme in different oxidation and ligation states at pH 7.5.** Spectra are shown for apo- (*gray*), monoheme (*red*), and diheme (*blue*) MhuD in the ferric state (*A*), as well as monoheme MhuD (*red*), diheme MhuD (*blue*), and free heme (*green*) in the ferrous (*B*) and ferrous-CO ligated (*C*) states. Spectra are normalized to the Soret peak.

The UV-vis electronic absorption spectra of ferric mono- and diheme MhuD at pH 7.5 are shown in Figure 3*A*. Monoheme has a Soret band maximum at 405 nm and is red-shifted from that of diheme at 396 nm. Both forms exhibit Q-bands at 562 nm, 586 nm, and 605 nm, the last of which is substantially more prominent in the diheme sample. For both forms, changes in pH had very minimal effect on the Soret and no changes in the positions of the Q-bands (Fig. S2 and Table S1). A more thorough discussion of UV-vis data for MhuD in the ferric state is provided in the Supporting information.

**Figure 4:**
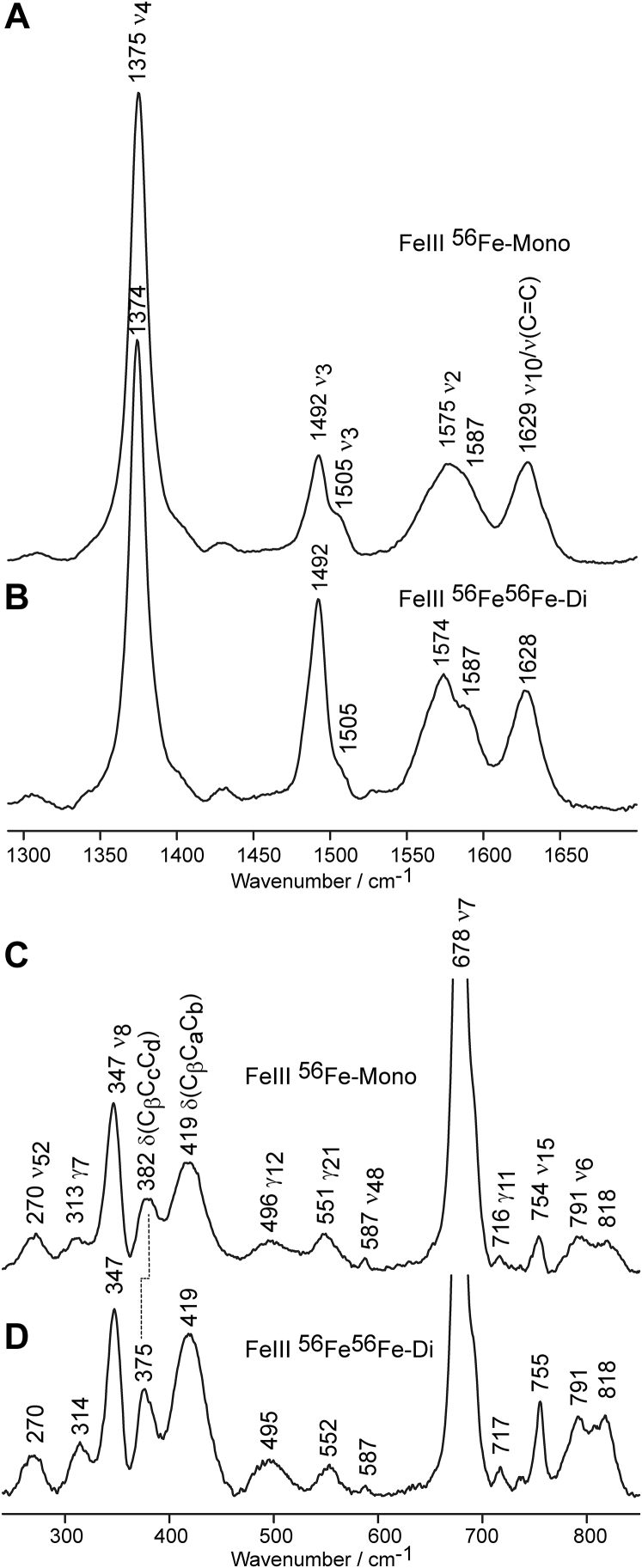
**rR spectra of mono- and diheme MhuD in the ferric state at pH 7.5.** Shown are the HF (*top*) and LF (*bottom*) regions of ferric monoheme (*A* and *C*) and diheme (*B* and *D*) MhuD measured with 406.7 nm excitation line at laser power of 10 mW.

The rR spectra of ferric mono- and diheme MhuD at pH 7.5 are shown in Figure 4. The vibrational modes were assigned based on data reported for HO-1, myoglobin, and heme model compounds (28, 29, 30, 31). The high-frequency (HF) spectra of mono- and diheme MhuD show spin state marker bands ν_3_ and ν_2_ at 1492 cm^−1^ and ∼1575 cm^−1^, respectively, indicating that both forms are predominantly 5-coordinated high spin (5cHS). A second set of ν_3_ and ν_2_ modes are observed at 1505 cm^−1^ and 1587 cm^−1^, respectively, associated with a minor contribution of 6-coordinated low spin (6cLS). The 6cLS state is most reasonably associated with the presence of a small fraction of heme iron coordinated to a water molecule that presumably has a hydroxide character caused by H-bonding interactions with the distal Asn-7 residue. The diheme spectrum shows an increase in relative intensity (with respect to ν_4_) of the 5cHS spin state markers, ν_3_ and ν_2_ at 1492 cm^−1^ and 1574 cm^−1^, respectively, while maintaining a similar relative intensity of the minor 6cLS state. This implies that the spin state of the His-coordinated heme remains the same in both the mono- and diheme active site, but the latter contains an additional 5-coordinated heme molecule. The rR spectra of ferric MhuD samples at different pH (Fig. S3 and Table S2) show that the 5cHS state remains dominant in acidic and alkaline conditions for both MhuD forms, contrary to the 6cHS H_2_O-bound to 6cLS OH^−^-bound transition observed in canonical HOs and IsdG/I proteins (28, 32). These results indicate that MhuD contains a predominantly hydrophobic distal active site environment.

The low-frequency (LF) region of rR spectra of heme proteins is rich with out-of-plane (oop) heme deformation modes, as well as modes associated with the heme peripheral groups and endogenous and exogenous axial ligands (21, 30, 31). Surprisingly, the LF spectra of ferric mono- and diheme samples differ only slightly, the main difference being the change in the propionate bending modes. Monoheme contains δ(C_β_-C_c_-C_d_) at 382 cm^−1^, and it shifts to 375 cm^−1^ in the diheme spectrum, implying that the propionate groups in monoheme form stronger H-bonds with one or more active site amino acids than those in diheme MhuD (33). This 7 cm^−1^ shift of δ(C_β_-C_c_-C_d_) illustrates the alteration of protein–heme interactions associated with the presence of the additional distal heme molecule, which reportedly induces a protein conformational change that almost triples the active site volume of diheme MhuD relative to its monoheme form (34).

Several out-of-plane (oop) modes are seen in the LF region, namely γ_7_ at 313 cm^−1^, γ_12_ at 496 cm^−1^, γ_21_ at 551 cm^−1^, and γ_11_ at 716 cm^−1^. Oop modes, especially those of B_1u_ symmetry such as γ_11_ and γ_12_, are expected to be more strongly activated for proteins with ruffled heme than those with planar heme (20, 35). Interestingly, no significant differences in activation of oop modes were detected between mono- and diheme MhuD, the latter of which actually has slightly greater enhancement of γ_7_ and γ_12_. This is surprising since previous crystallographic studies reported substantially different degrees of oop distortions of the hemes in mono- (1.4 Å) and diheme (0.7 Å) MhuD (10, 11).

### Ferrous state

The rR spectra of the ferrous state of mono- and diheme MhuD were measured using the 441.6 nm excitation line to exclusively enhance modes associated with the His-coordinated heme, while the other ferrous heme in the distal active site of diheme was effectively rR silent (Soret at ∼383 nm, Fig. 3*B*). The HF ferrous rR spectra are shown and discussed in Fig. S4.

**Figure 5:**
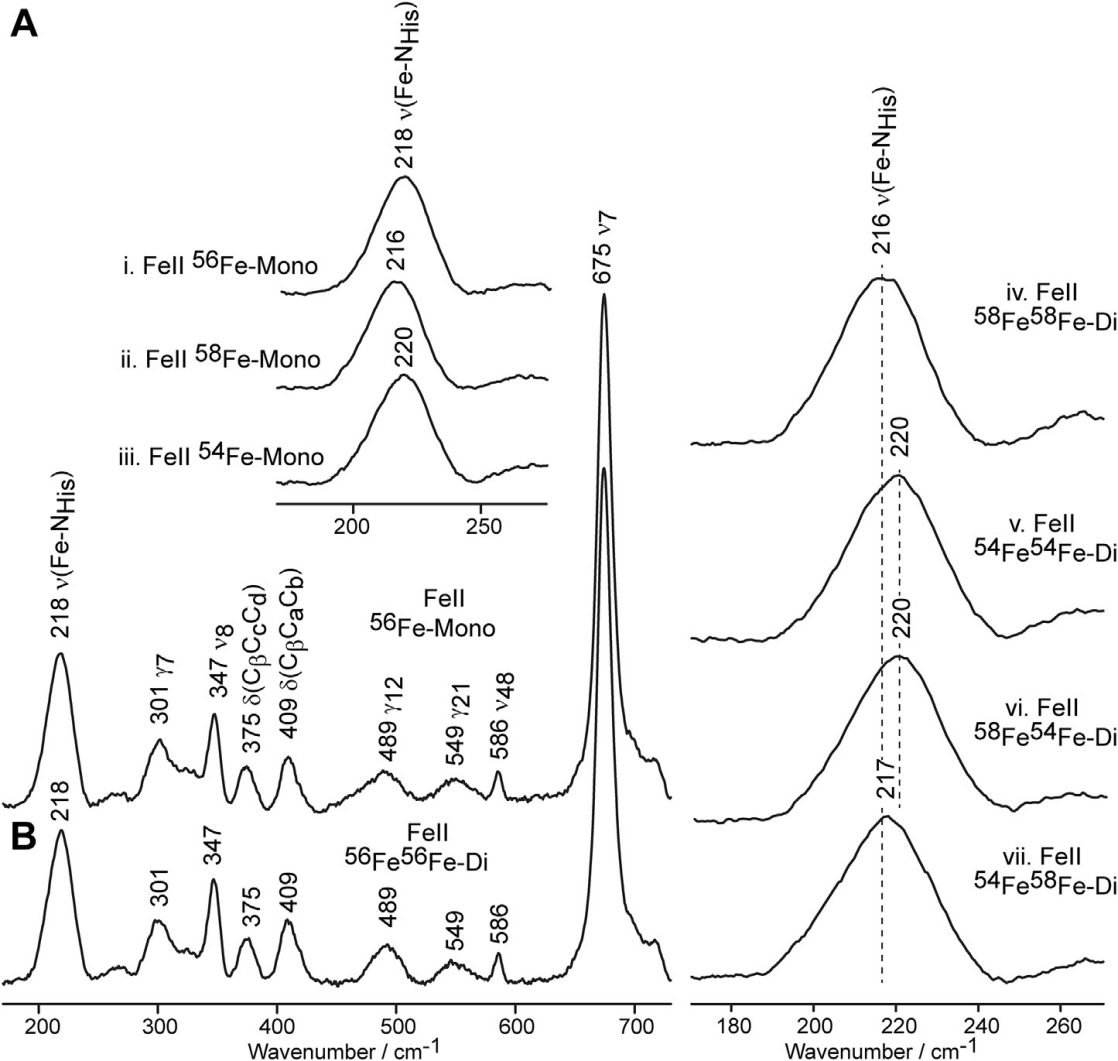
**rR spectra of mono- and diheme MhuD in the ferrous state.** Shown is the LF region of ^56^Fe-PPIX-bound monoheme (*A*) and diheme (*B*) MhuD at pH 7.5 measured with 441.6 nm excitation line at laser power of 10 mW. The ν(Fe-N_His_) mode is expanded for mono- (i–iii) and diheme (iv–vii) MhuD samples bound with different combinations of ^54^Fe and ^58^Fe isotopically labeled hemes.

Shown in Figure 5, the ν(Fe-N_His_) stretching mode of monoheme MhuD is seen at 218 cm^−1^, consistent with previously published data for monoheme MhuD and rat HO-1 (3, 29), and within 1 cm^−1^ of those of IsdG and IsdI (32). This frequency is indicative of neutral imidazole character of the proximal heme ligand, His-75. Interestingly, ν(Fe-N_His_) in the diheme spectrum is also seen at 218 cm^−1^, indicating that the presence of the distal heme in the active site does not affect the strength of the proximal heme Fe-N_His_ linkage. Additionally, as in ferric samples, no differences are observed in the degree of nonplanar heme deformations between mono- and diheme MhuD. The assignment of the ν(Fe-N_His_) stretching mode in the ferrous monoheme spectra was confirmed using isotopically labeled hemes ^54^Fe- and ^58^Fe-PPIX (Fig. 5, i–iii), which shifted the mode to 220 cm^−1^ and 216 cm^−1^, respectively. Likewise, ν(Fe-N_His_) is observed at 220 cm^−1^ and 216 cm^−1^ in the spectra of the ^54^Fe^54^Fe-diheme and ^58^Fe^58^Fe-diheme samples (Fig. 5, right), respectively, confirming the 4 cm^−1^ difference between these two iron isotopes is maintained in the spectra of diheme samples (full LF spectra are shown in Fig. S5).

The samples with mixed iron isotopes, ^58^Fe^54^Fe- and ^54^Fe^58^Fe-diheme, are named in the order of which the hemes were added to apoMhuD, *e.g.*, the ^58^Fe^54^Fe-diheme sample was prepared first as ferric ^58^Fe-monoheme and then excess ^54^Fe-PPIX was added to form diheme (see Experimental procedures). This strategy was employed to ensure that all samples were completely bound with one heme of a particular isotope prior to addition of the second and was necessary to facilitate reliable insight into the dynamics associated with binding of the second heme molecule in the same active site. The ^58^Fe^54^Fe-diheme sample (Fig. 5, right) exhibits the ν(Fe-N_His_) stretching mode at 220 cm^−1^, the same frequency as that of ^54^Fe-monoheme instead of the expected frequency of 216 cm^−1^, which would be observed if ^58^Fe-PPIX was still coordinated to His-75. This implies that the second incoming heme replaces the first as the His-ligated heme. The experiment was repeated for the ^54^Fe^58^Fe-diheme sample and ν(Fe-N_His_) was observed at 217 cm^−1^, roughly the same frequency as that of the ^58^Fe-monoheme sample, further confirming the His-coordinated heme switching mechanism during diheme MhuD formation.

### Ferrous-CO ligated state

The UV-vis spectrum of ferrous-CO monoheme MhuD exhibits a Soret maximum at 417 nm and the spectrum of diheme MhuD appears to have contributions of features observed in the CO-ligated free heme spectrum; *e.g.*, the Soret peak of free heme at 407 nm is at the same wavelength as the lower wavelength shoulder seen on the Soret band of the diheme MhuD spectrum (Fig. 3*C*).

The full range rR spectra of mono- and diheme MhuD samples, as well as free heme, are shown in Fig. S6 and discussed in Supporting information. The most notable differences in the spectra of mono- and diheme MhuD lie in the ∼500 to 600 cm^−1^ and 1800 to 2000 cm^−1^ regions, where the modes associated with the Fe-C-O fragment are typically observed for His-ligated proteins (26). Careful spectral deconvolutions in the LF and HF regions were performed as described in the Supporting information to determine the real frequencies of modes associated with the Fe-C-O fragments.

**Figure 6:**
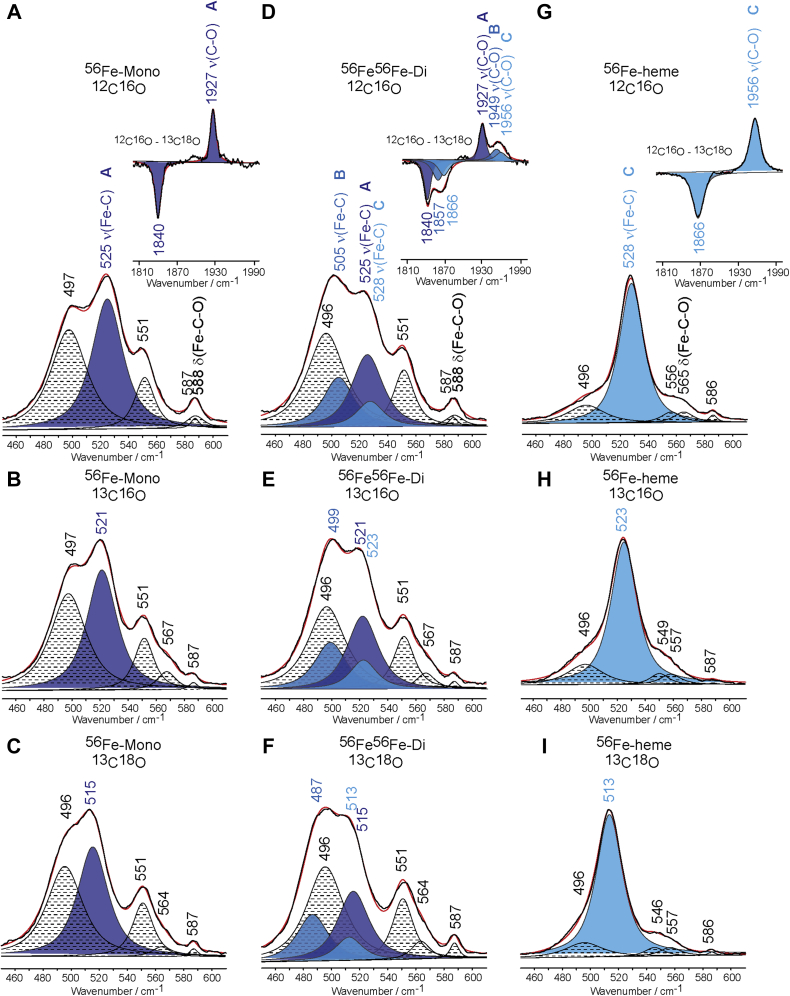
**Deconvoluted rR spectra of ferrous-CO adducts of mono- and diheme MhuD and free heme.** Shown is ^56^Fe-monoheme MhuD with ^12^C^16^O (*A*), ^13^C^16^O (*B*), ^13^C^18^O (*C*) isotopes, ^56^Fe^56^Fe-diheme MhuD with ^12^C^16^O (*D*), ^13^C^16^O (*E*), ^13^C^18^O (*F*) isotopes, and free ^56^Fe-PPIX with ^12^C^16^O (*G*), ^13^C^16^O (*H*), ^13^C^18^O (*I*) isotopes. The *insets* in (*A*), (*D*), and (*G*) show the ^12^C^16^O - ^13^C^18^O difference spectrum in the HF region for each respective sample. In each spectrum the *black line* represents the experimental data, and the *red line* shows the trace fitted with a mixed 75%/25% Lorentzian/Gaussian function. The *black* and *white* fitted peaks with *dotted lines* represent heme modes and δ(Fe-C-O) modes. The ν(Fe-C) and ν(C-O) modes for Fe-C-O conformers *A*–*C* are represented by *dark*, *medium*, and *light blue peaks*, respectively.

Shown in Figure 6 are the deconvoluted spectra for ^56^Fe-PPIX mono- and diheme MhuD and free heme samples in the 450 to 610 cm^−1^ range for ^12^C^16^O, ^13^C^16^O and ^13^C^18^O isotopes. The ν(Fe-C) stretching mode in the spectrum of monoheme MhuD is seen at 525 cm^−1^ for the naturally abundant isotopes, and it shifts to 521 cm^−1^ and 515 cm^−1^ upon substitution with ^13^C^16^O and ^13^C^18^O isotopes, respectively. The monoheme sample exhibits another isotopically sensitive mode at 588 cm^−1^, identified as the δ(Fe-C-O) bending mode, that shifts to 567 cm^−1^ for ^13^C^16^O and 564 cm^−1^ for ^13^C^18^O isotopes. The ^12^C^16^O – ^13^C^18^O difference spectrum in the 1800 to 2000 cm^−1^ region shows the ν(C-O) stretching mode at 1927 cm^−1^ for ^12^C^16^O that downshifts by 87 cm^−1^ for the heavier ^13^C^18^O isotopes. Deconvolution of the LF region also revealed the presence of additional heme modes at 497 cm^−1^, 551 cm^−1^, and 587 cm^−1^. The spectral parameters of these modes, such as their frequencies, bandwidths, and relative intensities, remained largely unchanged between the spectra of samples with isotopically labeled CO, as described in the Supporting information.

Interestingly, the deconvoluted spectra of diheme MhuD show multiple Fe-C-O conformers, denoted as A, B, and C, in both the LF and HF regions (Fig. 6, *D*–*F*). Conformers A and C have ν(Fe-C) and ν(C-O) modes located at the same frequencies and exhibit identical CO isotopic shifts and bandwidths as monoheme MhuD and free heme (Fig. 6, *G*–*I*), respectively (extracted data reported in Tables S3–S17). A third Fe-C-O conformer, conformer B, is unique to diheme MhuD and its ν(Fe-C) mode is seen at 505 cm^−1^ shifting to 499 cm^−1^ and 487 cm^−1^ upon ^13^C^16^O and ^13^C^18^O substitution, respectively. The corresponding ν(C-O) mode for conformer B is located at 1949 cm^−1^ and downshifts by 92 cm^−1^ with ^13^C^18^O isotopic replacement.

**Figure 7:**
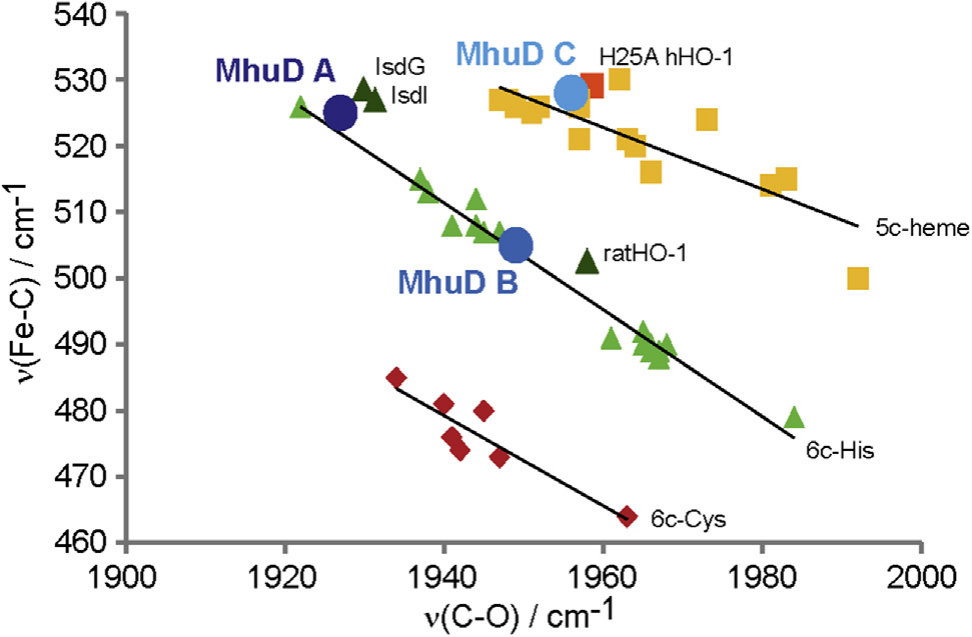
**Inverse correlation plot of ν(Fe-C) and ν(C-O) frequencies for ferrous-CO adducts of heme proteins.** Shown are MhuD conformers (*dark*, *medium*, and *light blue circles*), ratHO-1 (29), IsdG/I (*dark green triangles*) (32), H25A hHO-1 (*orange square*) (36), myoglobin variants (*light green triangles*) (26), and various cytochrome P450s (*red diamonds*) and heme model compounds (*yellow squares*) (27).

Shown in Figure 7 is an inverse correlation plot of ν(Fe-C) and ν(C-O) frequencies of MhuD and various other heme proteins and model compounds (26, 27, 29, 32, 36). The ν(Fe-C)/ν(C-O) pairs of MhuD conformers A and B fall on the line of 6-coordinated hemes with histidine as a proximal ligand, confirming the neutral character of the imidazole group of both conformers (26). Conformer A, which is present in both mono- and diheme MhuD, is located in the upper left region of the correlation line indicating strong positive polarization (26, 27), likely from H-bonding interactions with the Asn-7 residue. Similar interactions were seen between the CN^−^ ligand and Asn-7 in the ferric-CN MhuD crystal structure (11). Notably, conformer A lies on the correlation line remarkably close to the CO adducts of IsdG/I proteins that have Asn at very similar positions, implying a similar interaction for all three proteins (32). Since the distal asparagine residue is proposed to be responsible for directing the heme reactive oxygen species to the appropriate meso-carbons to form hydroxyheme in the catalytic mechanisms of MhuD and IsdG/I proteins, conformer A likely represents the configuration adopted by catalytically active Fe-O-O fragments (16, 17), implying diheme MhuD retains enzymatic activity. Conformer B of diheme MhuD lies toward the middle of the correlation line, closer to wild-type myoglobin and rat HO-1, which experience much weaker H-bonding interactions (26, 29). Conformer C falls on the correlation line characteristic of 5-coordinated heme model compounds, representing the CO-ligated heme in the distal active site, which is not ligated to the protein. Its position on the correlation plot is very similar to that of the H25A mutant of hHO-1 where the proximal heme ligand, histidine, was replaced with alanine, providing another example of a 5-coordinated species within a protein active site (36).

**Figure 8:**
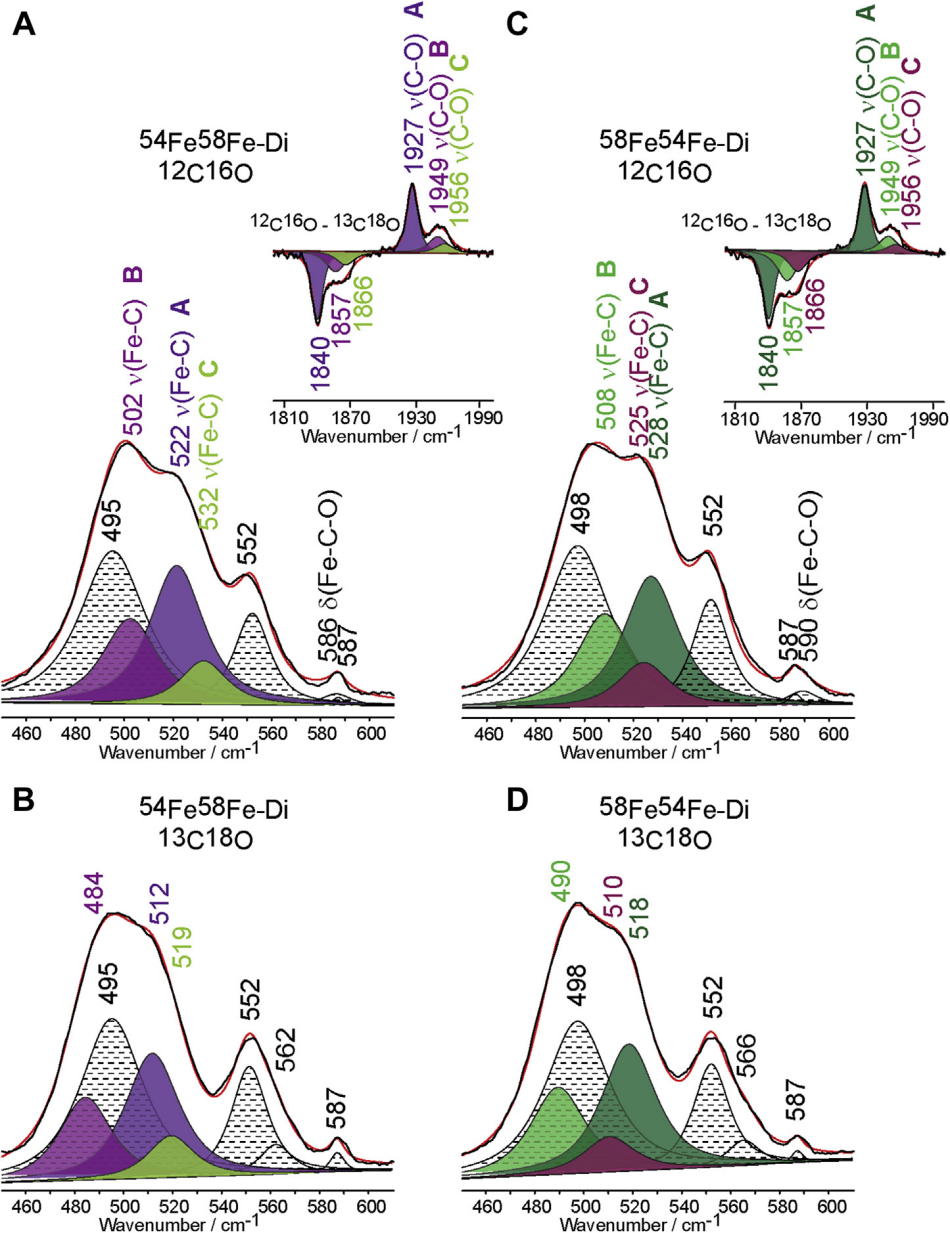
**Deconvoluted rR spectra of ferrous-CO adducts of diheme MhuD samples containing mixed heme Fe isotopes.** Shown are ^54^Fe^58^Fe-diheme MhuD with ^12^C^16^O (*A*) and ^13^C^18^O (*B*) isotopes, and ^58^Fe^54^Fe-diheme MhuD with ^12^C^16^O (*C*) and ^13^C^18^O (*D*) isotopes. The *insets* in (*A*) and (*C*) show the ^12^C^16^O - ^13^C^18^O difference spectrum in the HF region for each respective sample. For each spectrum, the *black line* represents the experimental data, and the *red line* represents the trace fitted with a mixed 75%/25% Lorentzian/Gaussian function. The *black* and *white* fitted peaks with *dotted lines* represent heme modes and δ(Fe-C-O) modes. The ν(Fe-C) and ν(C-O) modes are color-coordinated according to the isotope of heme iron, with ^54^Fe-CO conformers represented by *dark*, *medium*, and *light green peaks*, and ^58^Fe-CO conformers by *violet*, *magenta*, and *red peaks*.

The consistency of the deconvoluted spectral patterns of the Fe-C-O conformers was further confirmed using MhuD samples containing isotopically labeled hemes. The deconvoluted CO spectra of samples with ^54^Fe-PPIX are shown in Fig. S7 and ^58^Fe-PPIX in Fig. S8. For MhuD samples with mixed heme iron isotopes, ^54^Fe^58^Fe- and ^58^Fe^54^Fe-diheme, the deconvoluted spectra are shown in Figure 8. The ^54^Fe^58^Fe-diheme sample exhibits ν(Fe-C) modes associated with conformers A and B at 522 cm^−1^ and 502 cm^−1^, respectively, the same frequencies as those in the ^58^Fe^58^Fe-diheme sample. Conformer C, however, has ν(Fe-C) at 532 cm^−1^, the same frequency as that of the ^54^Fe^54^Fe-diheme sample, meaning the ^54^Fe-heme now occupies the distal active site. The analogous, but reverse case is observed for the ^58^Fe^54^Fe-diheme sample. These results reinforce the proposal derived from studies of the ferrous state (*vide supra*), that during formation of diheme MhuD, the incoming second heme replaces the originally His-coordinated heme and displaces it to the distal active site. It is noted that the reliability of the deconvolution methodology presented here is reinforced by the fact that the ratios of ν(Fe-C) peak areas between conformers A, B, and C in the spectra of diheme samples are consistent between each of the variations of CO and Fe isotopes (Tables S3–S17). The frequencies and isotopic shifts of ν(Fe-C), ν(C-O) and δ(Fe-C-O) for all samples measured herein are listed in Table 1.

**Table 1:** Data summary for ferrous-CO ligated mono- and diheme MhuD and free heme with isotopically labeled hemes

Sample	ν(Fe-C)^a^	ν(C-O)^a^	δ(Fe-C-O)^a^
^56^Fe-free	528 (5, 15)	1956 (44, 90)	565 (16, 19)
^56^Fe-mono	525 (4, 10)	1927 (43, 87)	588 (21, 24)
^56^Fe^56^Fe-di A	525 (4, 10)	1927 (43, 87)	588 (21, 24)
^56^Fe^56^Fe-di B	505 (6, 18)	1949 (43, 92)	
^56^Fe^56^Fe-di C	528 (5, 15)	1956 (44, 90)	
^54^Fe-free	532 (13)	1956 (90)	567 (19)
^54^Fe-mono	528 (10)	1927 (87)	590 (24)
^54^Fe^54^Fe-di A	528 (10)	1927 (87)	590 (24)
^54^Fe^54^Fe-di B	508 (18)	1949 (92)	
^54^Fe^54^Fe-di C	532 (13)	1956 (90)	
^58^Fe-free	525 (15)	1956 (90)	563 (19)
^58^Fe-mono	522 (10)	1927 (87)	586 (24)
^58^Fe^58^Fe-di A	522 (10)	1927 (87)	586 (24)
^58^Fe^58^Fe-di B	502 (18)	1949 (92)	
^58^Fe^58^Fe-di C	525 (15)	1956 (90)	
^54^Fe^58^Fe-di A	522 (10)	1927 (87)	586 (24)
^54^Fe^58^Fe-di B	502 (18)	1949 (92)	
^54^Fe^58^Fe-di C	532 (13)	1956 (92)	
^58^Fe^54^Fe-di A	528 (10)	1927 (87)	590 (24)
^58^Fe^54^Fe-di B	508 (18)	1949 (87)	
^58^Fe^54^Fe-di C	525 (15)	1956 (90)	

a Shown are the^12^C^16^O frequencies (cm^−1^) and in parentheses are the (Δ^13^C^16^O, Δ^13^C^18^O) or just (Δ^13^C^18^O) isotopic shifts (cm^−1^).

### Activity assays

**Figure 9:**
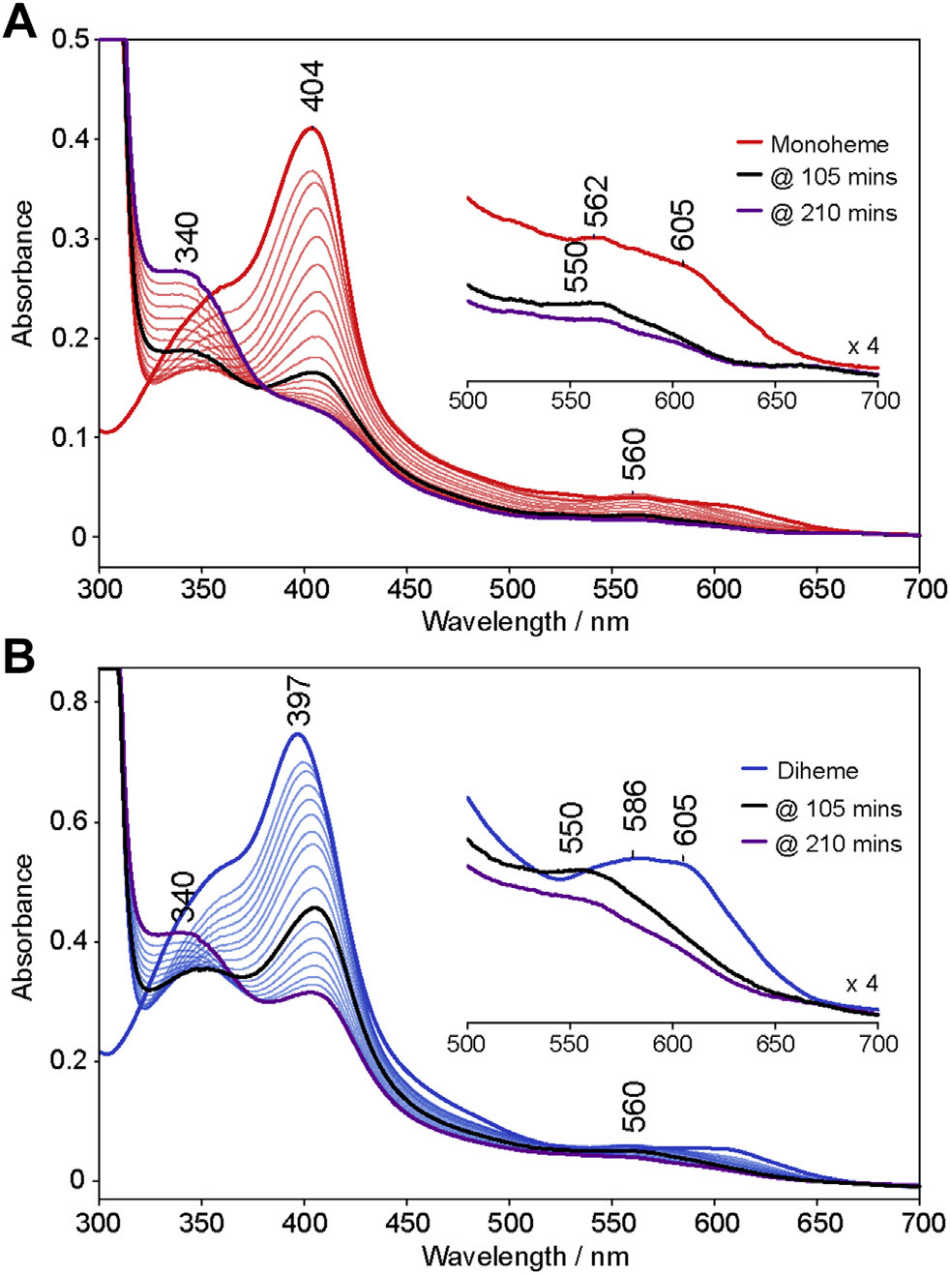
**UV-vis absorption spectra of ascorbate activity assays for mono- and diheme MhuD.** The monoheme (*A*) and diheme (*B*) MhuD assays were carried out at room temperature. Reaction mixtures contained 5 μM protein concentration of mono- or diheme MhuD in 100 mM potassium phosphate, pH 7.5 with 1250 units/ml catalase, 75 units/ml superoxide dismutase, 10 mM EDTA, and the reactions were initiated by addition of 10 mM sodium ascorbate.

In order to test the hypothesis that diheme MhuD is enzymatically active, several activity assays were performed and monitored by UV-vis spectroscopy. The natural redox partner for MhuD has yet to be identified, so the assays were carried out using electron donor substitutes, ascorbate (Fig. 9) and NADPH-cytochrome P450 oxidoreductase (POR, Fig. S9). The ascorbate assays were performed in the presence of superoxide dismutase and catalase to minimize nonenzymatic heme degradation, as confirmed by a control assay with free heme (Fig. S10). Shown in Figure 9, the absorbance of the Soret band diminishes over time for both mono- and diheme MhuD, indicating that heme is being degraded by both forms. As the reaction begins, both MhuD forms show an initial increase in the Q-band at ∼560 nm and shift of the Soret to ∼408 nm as oxy-MhuD accumulates, consistent with previously published ascorbate activity assays for monoheme MhuD (10, 11, 15, 17). This species decreases in absorbance as the reaction progresses and forms the products, mycobilins-a and b with bands at ∼340 nm and ∼550 nm. The absorbance spectrum of free mycobilin-a has bands at 345 nm and 565 nm, and those for mycobilin-b are at 336 nm and 555 nm (3). A plot of Soret Δabsorbance *versus* reaction time for mono- and diheme MhuD is shown in Fig. S11 and discussed in Supporting information. The activity of MhuD was consistent with different electron donors as POR also enabled heme degradation by both forms (Fig. S9), consistent with published POR assays for monoheme (3, 10), but contradictory to that reported for diheme MhuD (10). To ensure that the observed activity of diheme MhuD is not attributable to the presence of a small fraction of monoheme form generated during gel filtration, which could potentially strip one of the heme molecules from the diheme active site, an additional ascorbate assay of MhuD (4 μM) in the presence of a large excess of heme (20 μM) was performed (Fig. S12). It is clear that under these conditions MhuD still degrades all heme molecules to mycobilins, as evidenced by the decrease in the Soret band and increase in absorbance of the bands around 340 nm and 550 nm.

While it is well established that monoheme MhuD degrades heme to mycobilin products (3, 17, 37), the enzymatic activity of diheme MhuD reported here for the first time calls for characterization of its heme degradation products. The crude products of the ascorbate assay were analyzed by electrospray ionization–mass spectrometry (ESI-MS) and the data (Fig. S13) revealed that the major product of diheme MhuD catalysis is also mycobilin with m/z 611.25, like that of monoheme. A minor product with m/z 583.25 was detected at nearly identical relative abundance as previously reported data for the monoheme MhuD reaction and was identified as biliverdin (37). Therefore, the products of heme degradation by diheme MhuD are highly consistent with that of monoheme in that its major products are mycobilins.

## Discussion

### Structure–function correlation of mono- and diheme MhuD

rR spectroscopy was employed in this study to investigate several features of the active site environment that dictate the reactivity of mono- and diheme MhuD such as heme (non)planarity, hydrophobicity of the distal active site, strength of the Fe-proximal ligand bond, and active site amino acid interactions with heme distal ligands and peripheral groups. The rR data shown here provide for the first time a uniform picture of heme (non)planarity in mono- and diheme forms of MhuD, *i.e.*, the His-coordinated heme molecules in both forms have a similar extent of nonplanar deformations. Additionally, the ferric state spectra confirmed that MhuD has a largely hydrophobic distal active site that is void of an ordered water molecule network like that possessed by canonical HOs (13, 14), suggesting distinct modes of oxygen activation. The ferrous state rR spectra indicated that the strength of the Fe-N_His_ linkage, which has great implications for heme reactivity (23, 38), is unaffected by the presence of an additional heme in the active site, *i.e.*, it is the same for mono- and diheme forms. In fact, the ferrous rR spectra of both mono- and diheme are essentially identical, *e.g.*, there are no differences in activation of out-of-plane modes that would indicate varying degrees of heme ruffling or alterations to the geometry of heme peripheral groups. These data are important because they not only reflect a large and malleable active site of MhuD but imply that the His-coordinated heme might retain its inherent reactivity in both mono- and diheme forms.

While one can envision that binding of the second heme could be preceded by a conformational change of the protein structure to allow binding of the newly added heme on the distal side of the His-coordinated heme, a dramatically different pathway was revealed in the rR spectra of ferrous diheme samples containing mixed heme iron isotopes where the second heme was shown to replace the first as the His-coordinated species. This implies that binding of the second heme is preceded by a, perhaps allosteric, protein conformational change that prompts the cleavage of the original Fe-N_His_ bond and movement of the α2 helix to extend the proximal heme pocket and allow the now free His-75 residue to bind the incoming second heme while the original heme remains in the distal active site. Alternatively, the heme-binding mechanism could be a result of conformational selection due to a flexible active site and a relatively labile Fe-N_His_ bond. While the X-ray crystal structures displayed in Figure 2 show only one histidine-ligated state where His-75 acts as the sole endogenous heme ligand, there could be other conformational states where the heme is coordinated to alternative His residues. There are several histidines surrounding the MhuD active site; His-78, for example, is located only three residues away in a flexible loop region. Therefore, conformational selection would be plausible wherein the second incoming heme binds to one of the other histidine residues in a particular state either before, after, or simultaneously with the cleavage of the Fe-N_His_ bond of the original heme to ultimately displace it to the distal active site (with no endogenous ligand). Regardless of whether the heme displacement occurs *via* induced fit or conformational selection mechanisms, our data unambiguously reveal that this unusual heme switching process takes place. These results demonstrate the unique capability of rR spectroscopy to unveil subtle, but physiologically important biomolecular dynamics processes that are not easily assessed using other biophysical methods.

The rR spectra of ferrous-CO adducts presented here provide important new insight into the structural differences between mono- and diheme MhuD. Until now, it was not known if either of the heme molecules in the active site of the diheme protein is capable of binding exogenous ligands. More importantly however, the rR spectra revealed two His-coordinated Fe-C-O conformers in diheme MhuD, one of which retains H-bonding interactions with Asn-7, identical to that seen in monoheme MhuD (conformer A), and the second (conformer B) in which these interactions are significantly disrupted. The third Fe-C-O conformer in diheme MhuD, conformer C, is associated with the 5-coordinated heme that remains in the distal heme pocket and is intermittently positioned to alter the H-bonding interactions of conformer A to generate conformer B. It is also noted based on the inspection of relative intensity ratios of modes associated with conformers A and B, that the former is still dominant in diheme MhuD, indicating that the second heme does not completely block the access of the heme exogenous ligands to the key Asn-7 residue. This is, in fact, quite surprising because the crystal structure of diheme MhuD suggests that such interactions would be completely impeded by the presence of the distal heme in the active site (10). Such a stark contrast in the structural implications derived from the spectroscopic data collected in solution here as compared with that collected on crystalline forms is a clear indication of the inherent highly flexible nature of the MhuD heme pocket in which the heme molecules have more conformational mobility to maintain the interactions between the His-coordinated heme and the distal active site. In fact, careful inspection of the X-ray crystal structure for diheme MhuD (Fig. 2) shows that the Asn-7 residue is actually located above the edges of the heme macrocycle, rather than directly above the heme iron. Such positioning requires less dramatic movement of the distal heme to enable the interaction between Asn-7 and the His-ligated heme exogenous ligands. It is reasonable to assume that the diheme crystal structure captures only one of multiple conformational states present in solution, likely showing that which is associated with Fe-C-O conformer B where these interactions are blocked or weakened. Furthermore, the fact that diheme MhuD has a substantially large relative population of the catalytically predisposed conformer A apparently contradicts the proposal of the diheme protein being inactive, *i.e.*, the activity of diheme might be slightly diminished relative to monoheme, but not entirely lost.

### Catalytic activity

The data presented here revealed that both mono- and diheme forms of MhuD are enzymatically active. Not only does diheme MhuD degrade the His-coordinated heme, but also the distal heme in its active site as well. Interestingly, after degrading the bound heme molecules in its active site, MhuD proceeds to bind the excess heme present in solution and convert it to mycobilin (Fig. S12). Previous studies reported relatively high affinity of the protein for the products, suggesting that removal of mycobilins from the MhuD active site is likely mediated by another protein (34). Our data, however, indicate that such proteins are not required for successive heme degradation reactions, as discussed in detail in the Supporting information.

The catalytic activity of diheme MhuD shown here is in stark contrast with previously reported data (10). The main difference between the previous work and the studies presented here is that the former involved MhuD samples containing a His-tag on the C-terminus, whereas the protein in our study has no additional amino acids to the native MhuD sequence. In that work, the reasonable assumption was made that the His-tag would have a negligible effect on the properties and activity of MhuD, as is the case for most proteins. It seems, however, that for the particular case of MhuD, the presence of the His-tag does have a substantial effect on heme-binding properties and quaternary structure of the protein.

A recent study showed that the His-tag interfered with measurement of the dissociation constants for diheme MhuD, but not monoheme, resulting in a K_D_ disparity of three orders of magnitude between the tagged and tagless protein (18). Furthermore, it was also reported using size-exclusion chromatography that His-tagged diheme MhuD exists in various higher-order oligomeric states of the protein, while the monoheme form is strictly in dimeric state (39). When the diheme samples in our study were applied to a Sephadex G-75 column (GE Healthcare), no such separation of oligomeric states was observed. Similarly, analytical ultracentrifugation studies recently showed that diheme MhuD with the His-tag removed by TEV protease exists only as a dimeric protein (19). The aggregation of tagged diheme MhuD would likely impede access of electrons to heme that are required in the catalytic cycle and explain its apparent inactivity as reported previously. This is not an isolated case of a His-tag altering the activity and heme-binding affinity of heme proteins involved in the heme uptake and utilization pathways of pathogenic bacteria. Rv0203 is one such protein that, like MhuD, is involved in the heme uptake pathway of Mtb. The His-tag was shown to alter heme-binding affinity for Rv0203 (40). Another example is HupZ from *Streptococcus pyogenes* for which the His-tag induced heme stacking and higher-order oligomeric states of the protein that altered its enzymatic function (41).

### Functional implications

Since its discovery, the evolutionary significance of the capability for MhuD to bind either one or two hemes in the same active site has been theorized (10, 19, 39). It was proposed that diheme MhuD functions primarily as a heme storage protein during periods of high intracellular heme concentration to prevent iron-mediated toxicity, a function comparable to that of the iron storage role of Mtb ferritin proteins, BfrA and BfrB (10). Conversely, at times of low heme concentration, one of the heme molecules in the diheme active site could be released or transferred to other heme proteins to enable heme degradation by monoheme MhuD in order to harvest the iron for use in several cellular processes. While these proposals seem reasonable, it appears implausible in conditions with an influx of heme that MhuD would lose the functionality to degrade and actively decrease its intracellular levels and prevent cytotoxicity. The studies of IsdG/I may provide clues to the evolutionary significance of mono- and diheme forms of MhuD. IsdG/I both degrade heme to staphylobilins, presumably by the same enzymatic mechanism. However, IsdG has a secondary function not possessed by IsdI; it inhibits the ferrochelatase CpfC protein involved in the heme biosynthesis pathway of *S. aureus* (42). Thus, IsdG mediates the intracellular heme concentration by regulating the pathways of both heme biosynthesis and degradation. Perhaps the physiological roles displayed by the two separate *S. aureus* proteins, IsdG/I, are evolutionary features combined into one protein for Mtb; *i.e.*, monoheme MhuD, like IsdI, functions as strictly a heme degrader, whereas diheme functions analogously to IsdG as both a heme degrader and a regulatory protein to exert coupled control over heme degradation and biosynthesis pathways in Mtb.

## Experimental procedures

### Materials

^56^Fe-protoporphyrin IX (hemin-chloride) was purchased from Sigma-Aldrich and the isotopically labeled hemes, ^54^Fe- and ^58^Fe-protoporphyrin IX, from Frontier Scientific. BL21 (DE3) *E. coli* cells and chitin resin were obtained from New England Biolabs. Human NADPH-cytochrome P450 oxidoreductase (POR) was obtained from ProSpec, and human superoxide dismutase from Bio Basic. The ^13^C^16^O and ^13^C^18^O gas isotopes and all other materials, unless mentioned otherwise, were purchased from Sigma Aldrich.

### MhuD protein expression and purification

The use of cleavable inteins with affinity domains for protein purification has been successfully applied to several systems (43, 44, 45, 46, 47). The full-length MhuD protein was expressed and purified while fused at its C-terminus to a cleavable intein–DNA gyrase subunit A from *M. xenopi* (Mxe GyrA). The gene encoding the MhuD-Mxe GyrA fusion protein was ordered from ATUM in the pD454-SR vector containing a T7 promotor and a gene for ampicillin resistance. A transformation of the pD454-SR vector into BL21(DE3) *E. coli* cells was performed *via* heat shock method. Yeast extract tryptone (2xYT) media with 100 mg/l ampicillin was used for inoculation of expression cultures. Cells were grown at 37 °C while shaking at 220 rpm to an OD_600_ value of ∼0.6 before protein expression was induced by addition of 1 mM IPTG and temperature was lowered to ∼25 °C. The cells were harvested 4 h after inducing protein expression and the cell pellets were frozen at −80 °C.

The affinity purification using Mxe GyrA intein was performed according to procedures adapted from the IMPACT Kit (New England Biolabs). The cells were lysed by sonication on ice in 20 mM Tris-Cl, 50 mM NaCl, pH 8.5 with added DNAase, 1 mM PMSF, and 1 mM EDTA. Cell particulate was removed by centrifugation and lysate was applied to a column with chitin resin at 4 °C. The column was then washed with 20 mM Tris-Cl, 0.5 M NaCl, pH 8.5 to remove contaminating proteins, followed by equilibration with 20 mM Tris-Cl, 50 mM NaCl, 50 mM β-mercaptoethanol, pH 8.5, and stopping flow to induce intein thiolysis. Cleaved MhuD was eluted after incubating the proteins on the column for ∼24 h β-mercaptoethanol was removed from the eluted protein sample by buffer exchange into 100 mM borate, pH 9.1 before freezing at −80 °C.

### Reconstitution of apo-MhuD with heme: CN-CO replacement method

Hemin chloride was dissolved in 0.3 M NaOH and diluted to 500 μM with 100 mM borate, 50 mM KCN, pH 9.1, and pH was readjusted to 9.1 with HCl (48, 49). The hemin-dicyanide was added dropwise in fourfold molar excess to 100 μM apo-MhuD in 100 mM borate, 40 mM KCN, pH 9.1 and incubated overnight at 4 °C. The sample was then concentrated and applied to two successive Bio-Gel P6 columns (Bio-Rad) equilibrated with 100 mM borate, 20 mM KCN, pH 9.1, and 100 mM potassium phosphate, pH 7.5 (Raman buffer) to remove excess heme and cyanide, respectively.

The monoheme-CN MhuD sample was placed in a septum-sealed glass vial and degassed under carbon monoxide gas, while stirring, for 20 min before reduction by approximately threefold molar excess of freshly prepared sodium dithionite (50 mM) with a gas-tight syringe. CO gas continued to flow over the sample while stirring for an additional 5 min before transferring the sample to a glove box under argon atmosphere where it was applied anaerobically to a Bio-Gel P6 column equilibrated with Raman buffer saturated with CO gas to remove the excess dithionite and displaced cyanide. The ferrous-CO adduct of MhuD was then oxidized back to ferric state by addition of ∼100-fold molar excess of potassium ferricyanide, which was then removed on a Bio-Gel P6 column equilibrated with Raman buffer. The same method was applied to prepare monoheme MhuD samples containing the isotopically labeled hemes, ^54^Fe- or ^58^Fe-PPIX.

To generate diheme MhuD samples, a fivefold molar excess of hemin in 100 mM potassium phosphate, 30 mM caffeine, pH 7.5 was added to the ferric monoheme MhuD samples prepared using the CN-CO replacement method. Caffeine was used to prevent formation of heme dimers in the titration solution (50, 51, 52). Excess heme was removed on a Bio-Gel P6 column equilibrated with Raman buffer. The same method was employed to prepare diheme MhuD samples containing the isotopically labeled hemes. To generate the ^54^Fe^54^Fe- and ^58^Fe^54^Fe-diheme samples, ^54^Fe-PPIX was added in fivefold molar excess to the ferric ^54^Fe- and ^58^Fe-monoheme samples, respectively. Similarly, the ^58^Fe^58^Fe- and ^54^Fe^58^Fe-diheme samples were prepared by adding ^58^Fe-PPIX to the ferric ^58^Fe- and ^54^Fe-monoheme samples, respectively. The eluted protein samples were analyzed by Bradford and pyridine hemochrome assays to determine protein and heme concentrations, respectively, as described in detail in Supporting information.

### Preparation of samples for rR measurements

All MhuD samples for rR measurements were concentrated to ∼150 to 200 μM protein concentration in Raman buffer (100 mM potassium phosphate, pH 7.5). In total, 100 μl of each sample was transferred to a Wilmad 5 mm Economy NMR tube for rR measurements. The samples for ferric state studies at different pH were exchanged into the appropriate buffers: 100 mM potassium phosphate pH 5.5 or 100 mM borate pH 9.1. To prepare the ferrous and ferrous-CO samples, NMR tubes containing ferric samples in Raman buffer were septum-sealed to ensure an anaerobic environment. Samples were degassed under flow of argon for 25 to 30 min. For ferrous-CO samples, the NMR tubes were then filled with the appropriate isotopes of CO gas. The samples were then reduced by addition of approximately threefold molar excess of anaerobically prepared 50 mM sodium dithionite using a gas-tight syringe and rR spectra were measured immediately.

### Resonance Raman measurements

The ferric and Fe^2+^–CO adducts of MhuD were measured using the 406.7 nm and 413.1 nm excitation lines, respectively, obtained from an Innova 302C Kr^+^ laser (Coherent Inc). The ferrous MhuD samples were excited using the 441.6 nm laser line from an IK5351R-D He–Cd laser (Kimmon Koha). Scattered light was collected using a 1250M-Series II Spectrometer (Horiba Scientific) fitted with a Pylon:400B CCD detector (Princeton Instruments). The width of the entrance slit on the spectrometer was 150 μm and a grating with 1200 grooves/mm was used. The ferric and ferrous MhuD samples were measured using ∼10 mW laser power. The power on the Fe^2+^–CO MhuD samples was kept at 1 to 2 mW to prevent photodissociation of the CO ligand. The NMR tubes containing samples were spun throughout measurements to prevent localized heating, protein degradation, and photodissociation of CO adducts. Measurements were performed in the 180° backscattering geometry at room temperature using a cylindrical lens to focus the laser beam as a line image on the sample. Fenchone and acetone-D_6_ were used as standards for calibration, and spectra were processed using GRAMS/32 AI software (Galactic Industries).

### rR spectral deconvolution

The ν(Fe-C) stretching and δ(Fe-C-O) bending modes in the LF region of rR spectra of MhuD Fe^2+^–CO adducts overlap with several heme modes, which complicates the accurate determination of their frequencies. Similarly, the positions of the ν(C-O) stretching modes in the HF region are difficult to discern because they have relatively weak intensities. Therefore, in order to accurately determine the frequencies and extent of the isotopic shifts of these modes, the averaged spectra in the LF region, and difference patterns in the HF region were deconvoluted using a curve fitting procedure. The curve fitting application in GRAMS/32 AI software was employed, and the spectra were fitted with mixed 75%/25% Lorentzian/Gaussian functions. The overall peak fitting protocol was adapted from a previously published procedure (53) and is described in detail in Supporting information.

### UV-vis spectroscopy and activity assays

UV-vis electronic absorption spectra were measured between 200 and 800 nm with a 1.0 nm data interval and scan rate of 600 nm/min using an Agilent Cary 60 UV-Vis Spectrophotometer. Ferrous and ferrous-CO samples of MhuD, as well as free heme samples, were prepared by placing diluted ferric samples into septum-sealed quartz cuvettes and degassing them under flow of argon while stirring for 25 to 30 min. Samples were then reduced by titration with anaerobically prepared sodium dithionite until the Soret and Q-bands no longer changed in the ferrous spectra. Samples were then placed under flow of CO gas with stirring for 5 to 10 min before measuring the ferrous-CO spectra.

The ascorbate and POR activity assays were both performed in open air at ambient temperature (∼20 °C) in 10 mm pathlength quartz cuvettes. The ascorbate assays were performed with 5 μM mono- or diheme MhuD in 100 mM potassium phosphate, pH 7.5 with 1250 units/ml catalase, 75 units/ml superoxide dismutase, 10 mM EDTA, and the reactions were initiated by addition of 10 mM sodium ascorbate. Spectra were measured every 10 or 15 min for 3.5 h. The ascorbate assay of 10 μM free hemin-chloride was performed under the same conditions, and spectra were measured every 15 min for 2 h. The NADPH-cytochrome P450 oxidoreductase (POR) activity assays were performed with 5 μM mono- or diheme MhuD in 100 mM potassium phosphate, pH 7.5 with 1250 units/ml catalase, 75 units/ml superoxide dismutase, 10 mM EDTA, 100 μM NADPH, and reactions were initiated by addition of 50 nM POR. Spectra were measured intermittently over the course of 7 h.

### ESI-MS of heme degradation products

To analyze the crude products of heme degradation by MhuD samples, turnover reactions were performed with slight modifications to facilitate the reaction rates and minimize product decomposition prior to analysis. The reaction mixtures contained 10 μM mono- or diheme MhuD in 50 mM potassium phosphate pH 6.0 with 1500 units/ml catalase, 565 units/ml superoxide dismutase, 10 mM EDTA, and reactions were initiated by addition of 10 mM sodium ascorbate. Reactions were incubated at 37 °C and protected from light. After halting reactions by addition of HCl, the products were extracted into dichloromethane and washed three times with deionized water. The crude products were evaluated *via* ESI-MS. Samples were analyzed by direct injection and infused to the MS with 90% MeCN with 0.1% formic acid and 10% water with 0.1% formic acid (v/v) using a Thermo Vanquish high performance liquid chromatography system (HPLC). ESI-MS data were acquired using a Thermo Q-Exactive Orbitrap operated in full scan, positive ion mode, with a scan range of 560 to 640 m/z and 140,000 mass resolution. High-resolution MS data were analyzed using Xcalibur Qual Browser, where extracted ion currents (EICs) were obtained (using a 5 ppm mass tolerance) for m/z values corresponding to biliverdin (583.2541 m/z), mycobilin (611.2489 m/z), and heme (616.1766 m/z).

## Data availability

All data are contained within this article.

## Supporting information

This article contains supporting information (6, 10, 11, 14, 19, 28, 29, 30, 32, 34, 48, 49, 54, 55, 56, 57, 58, 59, 60, 61, 62, 63, 64, 65, 66, 67, 68).

## Conflict of interest

The authors declare that they have no conflicts of interest with the contents of this article.
