# Supplementary material for: Structure–function characterization of the mono- and diheme forms of MhuD, a noncanonical heme oxygenase from Mycobacterium tuberculosis
Source: J Biol Chem. 2021 Dec 6;298(1):101475. doi: 10.1016/j.jbc.2021.101475 (PMC8801480; doi:10.1016/j.jbc.2021.101475)
Supplement: Supporting information, Figures S1–S13 and Tables S1–S17 [file mmc1.pdf]

Supporting Information for

Structure-function characterization of the mono- and diheme forms of MhuD, a noncanonical heme oxygenase from *Mycobacterium tuberculosis*

Samuel N. Snyder[a], and Piotr J. Mak[a]*

[a]Department of Chemistry, Saint Louis University, Saint Louis, MO 63103 (USA)

Corresponding author*: Piotr J. Mak

Email: piotr.mak@slu.edu

Table of Contents

Supplementary experimental procedures (pp. S2-S3)

Supplementary results and discussion (pp. S3-S10)

Supplementary figures (pp. S11-S23)

Figure S1. SDS-PAGE gel image of MhuD purification using chitin resin. (p. S11)

Figure S2. UV-vis spectra of ferric mono- and diheme MhuD under different pH conditions. (p. S12)

Figure S3. rR spectra of ferric mono- and diheme MhuD under different pH conditions. (p. S13)

Figure S4. rR spectra in the HF region of ferrous MhuD. (p. S14)

Figure S5. rR spectra in the low frequency region of ferrous diheme MhuD samples with isotopically labeled hemes. (p. S15)

Figure S6. rR spectra of ferrous-$^{12}C^{16}O$ ligated MhuD and free heme. (p. S16)

Figure S7. Deconvoluted rR spectra of ferrous-CO adducts of ^{54}Fe MhuD and free heme samples. (p. S17)

Figure S8. Deconvoluted rR spectra of ferrous-CO adducts of ^{58}Fe MhuD and free heme samples. (p. S18)

Figure S9. UV-vis spectra of POR activity assays for MhuD. (p. S19)

Figure S10. UV-vis spectra of the ascorbate control assay for free heme. (p. S20)

Figure S11. Plot of Δabsorbance of the Soret peak over time for the ascorbate assays. (p. S21)

Figure S12. UV-vis spectra of ascorbate assay of MhuD with excess heme. (p. S22)

Figure S13. Electrospray ionization mass spectrometry (ESI-MS) spectrum of the crude products of heme degradation by diheme MhuD. (p. S23)

Supplementary tables (pp S24-S40)

Table S1. pH dependence of Soret and Q-band wavelengths in the UV-vis spectra of mono- and diheme MhuD, IsdG, IsdI, and rat HO-1 in the ferric state. (p. S24)

Table S2. Effect of pH on the frequencies of Raman shifts for spin state marker modes ν_2 and ν_3 for mono- and diheme MhuD, IsdG, IsdI, and rat HO-1 in the ferric state. (p. S25)

Tables S3-S17. Extracted ferrous-CO rR deconvolution data. (pp. S26-S40)

Supplementary experimental procedures

rR spectral deconvolution. The LF region, where the ν(Fe-C) and δ(Fe-C-O) modes can be observed, consists of heme modes that were necessary to include in the deconvoluted spectra to obtain a reasonable fit. To determine the position of the peaks associated with the heme modes, deconvolution of monoheme MhuD and free heme samples was performed first. The $^{12}C^{16}O$ - $^{13}C^{18}O$ difference patterns in the high frequency region revealed that there is only one isotope sensitive mode in these samples (Tables S3 and S4). This observation, along with the spectral response to isotopic CO substitution, allowed determination of which peaks in the LF spectra are associated with the heme modes and those associated with the ν(Fe-C) and δ(Fe-C-O) modes. The number of heme peaks were kept to a minimum. During iteration cycles between different CO isotopes, the intensities of the heme modes were allowed to change while their frequencies and bandwidths were restricted within ± 1.0 cm^{-1}. The intensities and frequencies of modes associated with CO were left unrestricted, while their bandwidths were restricted within ± 1.0 cm^{-1}. It is noted that the frequencies, intensities, and bandwidths of heme modes did not change significantly during the iteration cycles. The peaks associated with the ν(Fe-C) and δ(Fe-C-O) modes changed frequencies, while maintaining their relative intensities and bandwidths between spectra of different CO isotopes (Tables S5 and S6). This procedure provided sets of heme peak parameters that were used in deconvolution of diheme spectra of MhuD.

The deconvolution of diheme MhuD spectra was more complex due to presence of multiple Fe-C-O conformers. While initially it was difficult to confidently determine the amount of the Fe-C-O conformers from the LF spectra, the analysis of the HF region where the ν(C-O) modes can be observed, provided strong evidence for three conformers. Compared to the monoheme MhuD and free heme samples, each exhibiting just one ν(C-O) stretching mode, the difference patterns in the HF region of diheme samples clearly contained at least two ν(C-O) modes. One of the diheme conformers had ν(C-O) at the same frequency and bandwidth as that of monoheme MhuD. The second feature was at a frequency near to that of the free heme. Fitting this feature with only one mode with a frequency and bandwidth similar to that of the free heme samples failed to make acceptable fits to the experimental data. Instead, the satisfactory fits were obtained only when, besides modes with the frequencies similar to monoheme and free heme samples, an additional third mode was added whose frequency, bandwidth, and intensity were allowed to vary during the iteration procedure (Table S7). The low frequency region was deconvoluted using the parameters of heme modes derived from the spectra of monoheme MhuD samples. The frequencies and bandwidths of two ν(Fe-C) modes were restricted within ± 1.0 cm^{-1} of those of monoheme and free

heme samples, while their intensities were left unrestricted. A third ν(Fe-C) mode was added with its frequency, bandwidth and intensity left unrestricted for the $^{12}C^{16}O$ isotope (Table S8). This methodical approach produced spectral deconvolution data that revealed isotopic shifts that were in good agreement with theoretically predicted values, gave consistent relative intensities of the three Fe-C-O conformers in both low and high frequency regions, and provided high accuracies of the fits between simulated and experimental data across all diheme MhuD samples of different heme Fe and CO isotopes in the HF and LF regions. It is also noted that the frequencies, bandwidths, and relative intensities of the heme modes in the LF region did not change significantly between different CO isotopes, further confirming the reliability of the reported peak fitting procedure. The extracted frequencies, bandwidths, and peak areas for all deconvoluted spectra are summarized in Tables S3 – S17.

Supplementary results and discussion

Preparation of MhuD protein. New innovative methods were employed in preparation of MhuD protein samples in this study to ensure the most accurate model of the native protein. The expression of MhuD-Mxe GyrA fusion protein enabled affinity purification at high yields (~20 mg/L cell culture) and purity (>99%) in a single chromatographic step (Figure S1). The intein cleavage is preceded by a N→S acyl transfer between the side chain thiol and backbone amide of the N-terminal cysteine residue of Mxe GyrA adjacent to the C-terminal alanine residue (Ala-105) of MhuD. Then, addition of a thiol reducing agent induces intein thiolysis, cleaving Mxe GyrA in its entirety from MhuD and leaving a backbone thioester on Ala-105 that is quickly hydrolyzed to a carboxylic acid, yielding apoMhuD without any additional amino acids to the native protein primary structure.

A novel reconstitution method was utilized in this study to generate samples of pure mono- and diheme forms of MhuD protein bound with proper, stoichiometric amounts of heme in each active site. It was recently shown for MhuD protein with the His-tag removed by TEV protease that a fraction of the sample forms diheme even when apo-MhuD is reconstituted with hemin-chloride in a 1:1 molar ratio (19). Additionally, while working with a protein that can bind two heme molecules in one active site, it should be taken into consideration that free heme tends to form μ-oxo and π-stacked dimers in aqueous solution (49, 54, 55). Thus, it is reasonable to envision that μ-oxo dimers could bind in the same active site during the reconstitution process, preventing formation of pure monoheme samples. Reconstitution in this study by the CN-CO replacement method provides an apparent solution to this challenging problem. Hemin-dicyanide exists as a

monomer in aqueous solution (48, 49), and our studies show that MhuD is incapable of *sequentially* binding two hemin-dicyanide molecules in the same active site. In order to remove the strong field CN^- ligand and generate ferric monoheme MhuD samples, additional steps were required; e.g., reduction of the ferric-CN adducts under CO atmosphere, chromatographic removal of displaced CN- under anaerobic conditions, and finally oxidation of CO adducts using potassium ferricyanide. The effectiveness of this method in producing pure monoheme samples without contamination by residual diheme populations is evidenced by consistent formation of samples with a roughly 1:1 heme:protein molar ratio despite being incubated with ~4-fold excess of hemin-dicyanide. An excess of heme was then added to the ferric monoheme samples, rather than apoprotein, to generate pure diheme MhuD samples. This was done to effectively monitor the effects that the distal heme has on the His-coordinated heme as the diheme samples were generated by addition of another equivalent of heme to the same monoheme samples that they are being compared to. This also ensured sequential binding of the second heme that converts mono- to diheme form. For such obtained mono- and diheme MhuD samples bound with various combinations of isotopically labeled hemes, the protein and heme concentrations were verified by Bradford and pyridine hemochrome assays, respectively (56). The average heme:protein ratio of 0.95:1.00 was determined for monoheme samples and 2.05:1.00 for diheme samples.

Ferric state of MhuD at different pH. The effect of pH on the UV-vis spectral pattern of mono- and diheme MhuD in Figure S2 shows that from acidic (pH 5.5) to near neutral (pH 7.5) to basic (pH 9.1) conditions, the Soret of monoheme shifts from 402 nm to 405 nm to 398 nm respectively, while the diheme Soret exhibits much smaller shifts (1-2 nm) in the corresponding pH range. There are no changes in the positions of the Q-bands for either form of MhuD in response to pH. The UV-vis spectra of mono- and diheme forms of MhuD are different from those of both canonical mammalian HO-1 and the noncanonical homologs–IsdG and IsdI. The Soret band of canonical rat HO-1 shifts from 404 nm at acidic to 413 nm at alkaline pH, reflecting a conversion from water-bound 6cHS state in acidic conditions to hydroxide-bound 6cLS in alkaline pH (28,29,57). This transition is also accompanied by shifts of the Q-bands from 500 and 631 nm at acidic pH to 540 and 575 nm at alkaline pH (57). The IsdG and IsdI proteins undergo a similar pH-dependent spin state conversion to HO-1, although their absorption patterns are different. This was attributed to the fact that IsdG/I proteins have strongly ruffled heme, whereas canonical HO has relatively planar heme (32). It was shown, however, through EPR and rR spectroscopies, that at neutral and basic pH, the hemes in these two proteins were 6-coordinated with hydroxide as the distal ligand (32). While HO-1 and IsdG/I display distinctive spectral changes when forming the 6-coordinated state with a distal hydroxide ligand at alkaline conditions, both mono- and diheme

forms of alkaline MhuD do not differ significantly from those at acidic or neutral conditions. The wavelengths of Soret and Q-bands of MhuD, IsdG, IsdI, and rat HO-1 at different pH conditions are summarized in Table S1.

The rR spectra of ferric mono- and diheme MhuD at different pH are shown in Figure S3. The high frequency (HF) region of monoheme MhuD confirms that the 5cHS state is dominant under acidic, neutral, and basic pH conditions. At pH 5.5 and 7.5, the ratio of relative intensities of ν_3 modes characteristic of 5cHS and 6cLS states remains relatively unchanged; however, at pH 9.1, the relative intensity of the 5cHS state increases. The effect of pH on the high frequency region for diheme MhuD samples are even more subtle, the main difference being a slight increase in the intensity of the ν_3 mode at 1492 cm^{-1} when pH increases. Similarly, the rR spectra in the low frequency region for both mono- and diheme MhuD show rather minor changes as a function of pH. The frequency of the $\delta(C_\beta\text{-}C_c\text{-}C_d)$ propionate bending mode indicates moderately weaker interactions in alkaline conditions and stronger H-bonding interactions at acidic conditions for both MhuD forms. The $\nu(Fe\text{-}OH_2)/\nu(Fe\text{-}OH)$ stretching modes associated with the water/hydroxide ligands are typically found in the ~490-560 cm^{-1} range (29, 32, 58). While there is a slight increase in the relative intensity of the 551 cm^{-1} band as pH increases for both mono- and diheme (Figure S3, left), no shifts of this mode or any others in this region were observed for the measurements in D_2O solvent or with isotopically labeled hemes (data not shown); i.e., there is no evidence of $\nu(Fe\text{-}OH_2)/\nu(Fe\text{-}OH)$ stretching modes in MhuD spectra. It is noted that the rR data presented here for MhuD at different pH are quite distinct from those of canonical HO-1 and the homologous noncanonical HO enzymes. The ν_3 and ν_2 modes in the spectra of mammalian HO-1 shift from 1483 cm^{-1} and 1565 cm^{-1} at acidic pH to 1503 cm^{-1} and 1582 cm^{-1} at alkaline pH, respectively (28), consistent with a full spin state conversion from 6cHS to 6cLS form and formation of the ferric hydroxide with the $\nu(Fe\text{-}OH)$ seen at 546 cm^{-1} (29). The spin state marker modes of IsdG/I undergo more complicated pH-induced changes, but formation of the hydroxide complex at basic pH was confirmed by detection of the $\nu(Fe\text{-}OH)$ stretching mode at 544 cm^{-1} for both IsdG and IsdI (32). The frequencies of ν_2 and ν_3 for MhuD, IsdG, IsdI, and rat HO-1 are summarized in Table S2.

In support of previously published crystal structure data (10, 11), the rR data reported here indicate that the distal active site of MhuD is hydrophobic, lacking a distal water network like that observed for canonical HO (14, 59). Notably, the high frequency region of monoheme MhuD at pH 7.5 closely resembles ferric rR spectra of the G139L mutant of human HO-1 which has a highly disturbed distal water network and exhibits a similar predominantly 5cHS heme with a minor population of 6cLS state (60). The subtle differences in the rR spectra of MhuD as a result of pH in Figure S3 are attributed to alterations of the heme active site environment resulting from

conformational changes of the protein structure; such protein flexibility is not uncommon. MhuD can adopt four conformational states of varying active site volume depending on the presence of heme or products: apo (no crystal structure available), monoheme (~190 Å^3), diheme (~530 Å^3), and product-bound (~360 Å^3) (34). The product-bound crystal structure was determined for the R26S-MhuD bound with biliverdin, which is the major heme degradation product of this mutant. It shows formation of a new protein structural feature, α-helix 3, which was suggested by molecular dynamics simulations to be also present in the product-bound wild-type protein as well. Furthermore, the electrostatic molecular surface of the active site undergoes alterations during the conformational transition from monoheme to product-bound forms, making it reasonable to envison similar types of changes to the active site environment of the highly flexible MhuD protein in response to pH alteration.

Ferrous state of MhuD. Figure 3 (B) in the main text shows the UV-vis spectra of mono- and diheme MhuD, as well as free heme (not ligated to protein) in the ferrous state. The maxima of the Soret bands, λ_{max}, are at 429 nm for monoheme and 425 nm for diheme MhuD. The spectrum of the diheme sample exhibits a shoulder at ~383 nm which coincides with the position of the Soret λ_{max} of free heme in the ferrous state. The Q-band regions of mono- and diheme samples both contain a broad band centered at 556 nm, and the diheme spectrum has a shoulder around ~575 nm that resembles a contribution from the Q-band region of free heme. The UV-vis spectra of ferrous IsdG and IsdI have Soret maxima at 413 nm and 424 nm, respectively, and both have a Q-band around 556 nm (32). The spectrum of ferrous mammalian HO-1 has Soret and Q-bands at the same wavelengths as monoheme MhuD (28).

Figure S4 shows the ferrous state rR spectra in the HF region of mono- and diheme MhuD bound with the naturally abundant isotope of heme Fe, ^{56}Fe-protoporphyrin IX (PPIX). The high frequency region of both mono- and diheme MhuD samples are virtually identical and exhibit oxidation and spin state marker bands ν_4 at 1355 cm^{-1}, ν_3 at 1470 cm^{-1}, and ν_2 at 1560 cm^{-1}, respectively, which are consistent with the previously published rR spectra of ferrous 5-coordinated heme of mammalian HO-1 (29), and are in close proximity to the values reported for ferrous IsdG/I where the ν_4, ν_3 and ν_2 modes are found at 1352/1353 cm^{-1}, 1478/1474 cm^{-1} and 1560/1559 cm^{-1}, respectively (32). The degree of similarity between rR spectra in HF (Figure S4) and LF (Figure 5, main text) regions of ferrous mono- and diheme MhuD samples confirms that when measured with 441.6 nm excitation line, the distal heme in the diheme active site (Soret at 383 nm) is effectively rR silent in the ferrous state despite its obvious presence in the heme pocket of diheme samples evidenced by UV-vis spectral differences from monoheme (Figure 3 B, main text). Since there is selective rR enhancement of only modes associated with the His-coordinated heme, its structure

and axial Fe-N_{His} linkage strength observed in monoheme MhuD are unaffected by the presence of the distal heme in the diheme form, as indicated by the rR data presented in this study.

Shown in Figure S5 is the full spectra in the ~200-700 cm^{-1} range of the low frequency (LF) region of ferrous diheme MhuD samples bound with different combinations of isotopically labeled hemes to supplement the data in Figure 5 of the main text, in which only a closeup of the ν(Fe-N_{His}) stretching mode is shown. With the exception of ν(Fe-N_{His}), no iron-isotope sensitive shifts are observed for the modes in the full LF spectra of these samples. There are only minor differences in the relative intensity of some modes between samples which are likely attributed to differences in the baselines in the spectra.

Ferrous-CO ligated state of MhuD. Figure 3 (C) in the main text shows the UV-vis spectra of ferrous-CO adducts of mono- and diheme MhuD, as well as free heme. Mono- and diheme MhuD have Soret peaks at 417 and 416 nm, respectively, and both contain Q-bands at ~537 nm and ~565 nm, the latter having greater absorbance in the diheme sample than monoheme. The spectrum of diheme MhuD appears to have contributions of features observed in the CO-ligated free heme spectrum, e.g., the shoulder on the blue side of the diheme Soret peak is at ~407 nm, the same wavelength as the Soret peak of free heme. Similarly, the Q-band around 565 nm in the spectrum of free heme has higher absorbance which could explain the increased absorbance of this band in the diheme MhuD spectrum. The UV-vis data imply that monoheme MhuD contains a 6-coordinated CO-heme bound to the proximal His-75 residue, while diheme MhuD has both a 6-coordinated and 5-coordinated CO species, the latter showing similarity to the 5-coordinated free heme CO adduct. The diheme spectrum also contains a Q-band at 632 nm that is absent from the other two samples, providing evidence for the possibility of an additional Fe-C-O conformer unique to the diheme form of MhuD.

The full rR spectra in the ~300-750 cm^{-1} and ~1300-1700 cm^{-1} regions for ferrous-CO adducts of ^{56}Fe-PPIX-bound mono- and diheme MhuD, as well as free heme samples are shown in Figure S6 to supplement the data in Figure 6 of the main text, which only shows the 450-610 cm^{-1} and 1800-2000 cm^{-1} regions. In Figure S6, the high frequency region of the monoheme spectrum shows the ν_4 oxidation state marker at 1375 cm^{-1} and spin state markers ν_3 at 1494 cm^{-1} and ν_2 at 1577 cm^{-1}. The diheme sample shows an upshift of the spin state marker bands to higher frequencies, with ν_3 and ν_2 modes at 1498 cm^{-1} and 1583 cm^{-1}, respectively. The frequencies of the oxidation and spin state markers for mono- and diheme MhuD are in good agreement with the expected frequencies of these modes in 6cLS ferrous-CO adducts of other His-ligated heme proteins (29, 61). The rR spectrum of the ferrous-CO adduct of rat HO-1 has ν_4, ν_3 and ν_2 modes at 1372 cm^{-1}, 1497 cm^{-1} and 1583 cm^{-1}, respectively (29). Interestingly, these values are different from the data

reported for the CO-ligated forms of the homologous noncanonical heme oxygenases, IsdG and IsdI, which have ν_4 at 1372 cm^{-1}, but the ν_3 and ν_2 modes were downshifted by ~20 cm^{-1} each from the typical ranges observed for His-ligated 6cLS CO adducts (32), a disparity attributed to the extensive ruffling of the heme macrocycle in both proteins. It is noted that the frequencies of ν_3 and ν_2 modes in the spectrum of diheme MhuD are apparently upshifted, implying contributions from other Fe-C-O species, an observation consistent with UV-vis data discussed above.

In the low frequency region of Figure S6, the ν_7 mode in the diheme MhuD spectrum is seen at 677 cm^{-1}, about halfway between that of monoheme (679 cm^{-1}) and free heme (676 cm^{-1}) samples. The propionate bending mode $\delta(C_\beta\text{-}C_c\text{-}C_d)$ is at the same frequency (382 cm^{-1}) for both mono- and diheme samples but has larger relative intensity in the monoheme spectrum. The band at 396 cm^{-1}, which is likely associated with the $\delta(C_\beta\text{-}Me)$ methyl bending mode, has better resolution in the monoheme spectrum than diheme. Both samples contain $\delta(C_\beta\text{-}C_a\text{-}C_b)$ vinyl bending modes at 415 and 425 cm^{-1}, however, the latter exhibits slightly stronger enhancement in monoheme, while the former is more enhanced in the diheme sample. The $\delta(C_\beta\text{-}C_a\text{-}C_b)$ bending modes appear in the range of ~400-440 cm^{-1} and the lower and higher frequencies indicate ip and oop orientations of the vinyl groups, respectively (62, 63); i.e., the presented data indicate that the geometry of the vinyl groups on one or both hemes in the diheme active site slightly favor a more ip orientation relative to the vinyl groups in the monoheme form. It is also noted that the spectrum of the monoheme sample exhibits stronger enhancement of the band at 359 cm^{-1}, a region where the oop γ_6 mode or a propionate bending mode with weak H-bonding interactions are typically observed (30, 64). While it is possible that activation of this oop mode in the monoheme spectrum might indicate stronger deformation of the heme compared to the His-ligated heme in the diheme sample, the lack of enhancement of other oop modes (e.g., γ_7 mode) argues against such hypothesis. Instead, it is more likely that the 359 cm^{-1} mode represents weak H-bonding interactions experienced by one of the heme propionate groups in monoheme MhuD, which are further destabilized by the presence of an additional heme in the diheme form.

Ferrous-CO rR spectra were also measured and deconvoluted for MhuD and free heme samples with isotopically labeled hemes (Figures S7-S8, and Figure 8 in main text) which confirmed the reliability of the deconvolution methodology employed in this study, showing that the ratios of ν(Fe-C) peak areas between conformers A, B and C in the spectra of diheme samples are consistent between each of the variations of Fe and CO isotopes (data in Tables S3-S17). The average relative peak area ratios of ν(Fe-C) modes of conformers B and C to the most prominent conformer A are 0.684:1.000 with a standard deviation (SD) of 0.085 and 0.286:1.000 with SD of 0.022, respectively. Likewise, the ν(C-O) stretching modes associated with the three conformers

show similar relative intensity ratios, with conformers A and C having the largest and the smallest peak areas, respectively.

Heme degradation activity assays and ESI-MS product analysis. Non-enzymatic heme degradation is known to occur in activity assays with ascorbate through the process of coupled oxidation (6, 65, 66). Myoglobin and hemoglobin, which have no heme degrading activity in vivo, will degrade heme to α-biliverdin in the presence of ascorbate (65, 66). Catalase and superoxide dismutase are scavengers for H_2O_2 and oxygen radicals, respectively, and are used to minimize the coupled oxidation pathway. An ascorbate control assay, shown in Figure S10, was performed on a free heme sample without protein under the same conditions as the mono- and diheme MhuD assays over the course of 2 hours to ensure the mitigation of coupled oxidation. The spectral changes observed over time were negligible when compared to those of mono- and diheme MhuD, confirming that the use of catalase and superoxide dismutase effectively minimized coupled oxidation.

Figure S11 monitors the progress of the heme degradation reactions for mono- and diheme MhuD in the ascorbate activity assays from Figure 9 in the main text. Since the rate at which Soret absorbance decreases is proportional to the rate of heme degradation, Figure S11 shows that the mono- and diheme samples degrade heme at roughly the same rate for the first 60 minutes, which is contradictory to the implications derived from the rR spectra of CO adducts which suggested lower activity or rates of heme degradation in diheme MhuD as compared to monoheme. Namely, assuming similar Raman cross-sections for modes associated with both A and B conformers, the conformer A that interacts with the catalytically important Asn-7 residue constitutes only ~65% of the overall Fe-C-O population in diheme samples, implying ~35% lower activity. Furthermore, one could propose that diheme conformer B represents an orientation that can still be enzymatically active but hydroxylates heme without regioselectivity. However, the regiospecificity of heme degradation was confirmed by the absorption spectral patterns (Figure 9, main text) and ESI-MS (Figure S13) of the products, which were identical for both mono- and diheme MhuD samples. A more reasonable explanation for the similar reaction rates of mono- and diheme throughout the initial 60 minutes is that ascorbate is inefficient at donating electrons to the catalytic cycle and effectively introduces a new rate-limiting step to the enzymatic reaction. The oxidoreductase protein, IruO has been proposed as a potential in vivo redox partner of IsdI and IsdG proteins (67, 68), and it was shown that activity assays carried out for IsdI/G had ~10-fold greater rates of heme degradation in the presence of IruO and NADPH than those in the presence of ascorbic acid (68). It is reasonable to assume that the reaction for MhuD in the presence of its natural redox partner

would also be significantly faster than ascorbate, and a divergence in the reaction rates of mono- and diheme MhuD would likely be observed.

At approximately 75 minutes the monoheme reaction starts to plateau as it has degraded most of its heme, while the diheme reaction continues at a similar rate as it apparently begins to degrade the second heme bound in its active site. It has been suggested that due to the relatively high affinity of the protein for the products, removal of mycobilins from the MhuD active site is likely mediated by another protein, although the model for the product affinity was based on biliverdin-bound MhuD instead of mycobilin (34). However, the data presented here suggests that the second heme is degraded in the absence of such a protein. The previously proposed affinity of MhuD for heme is still ~6-fold greater than biliverdin, and potentially more disparate for mycobilin (34). Therefore, we propose that the His-bound heme is converted to mycobilin which is then displaced by the heme from the distal active site and subsequently released from the heme pocket due to lesser affinity for mycobilin than heme in the proximal active site. Such a replacement of the porphyrin species and their derivatives in the proximal active site seems plausible considering our data for the process of heme binding displayed for diheme MhuD, albeit in reverse order.

Supplementary figures

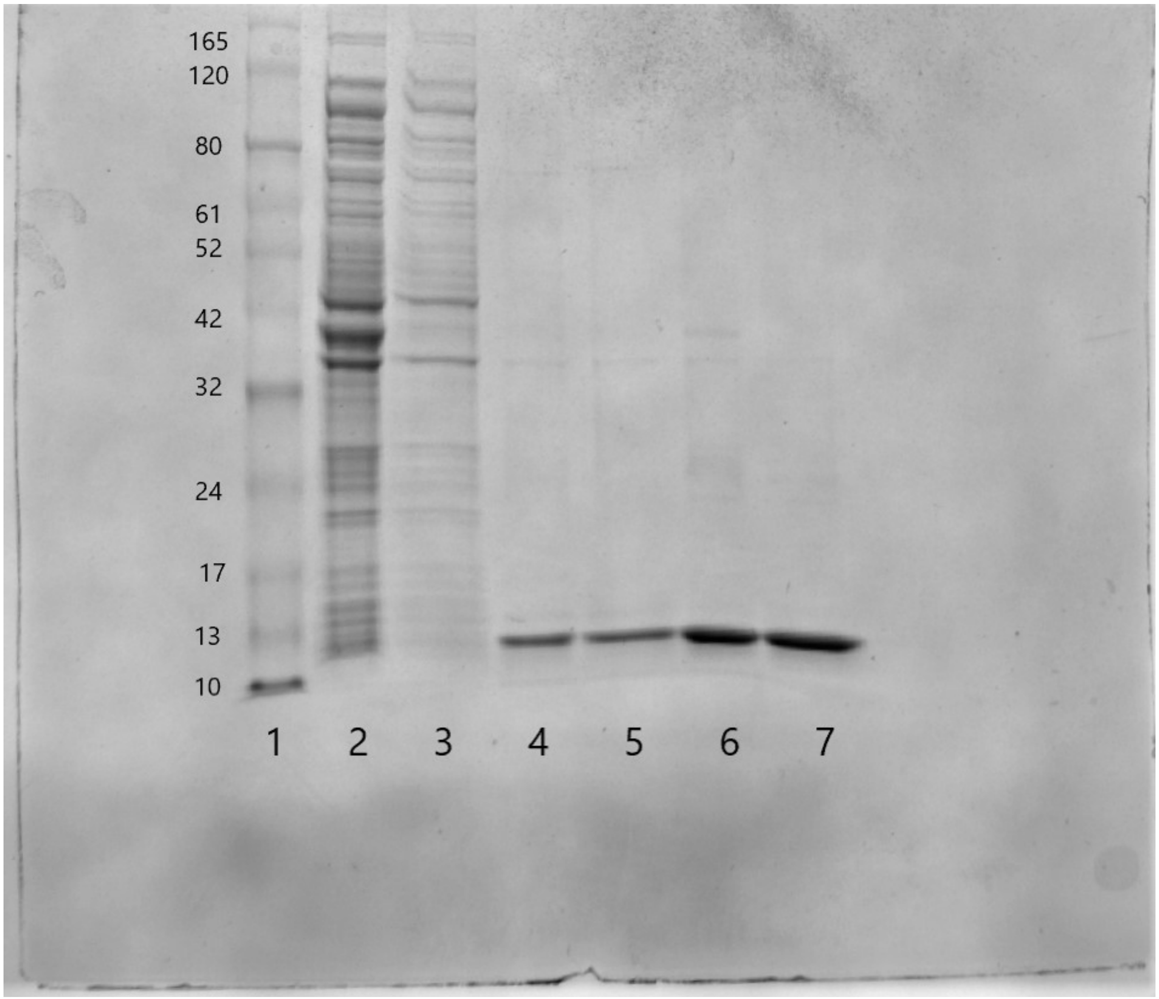

Figure S1. SDS-PAGE gel image of MhuD purification using chitin resin. Samples were heated in SDS sample loading buffer without β-mercaptoethanol (β-ME) prior to applying to bis-tris ExpressPlus PAGE Gels (GenScript) gels. β-ME was omitted to identify any uncleaved MhuD-fusion protein eluted from the column, i.e., to ensure that cleavage of the MhuD-intein fusion proteins occurred on the column and not just in the heated sample loading buffer. Lane 1 is the pre-stained 10-175 kDa Wide Range Protein Ladder (Bio Basic) with molecular weight markers labeled in kDa. Lane 2 is the clarified cell lysate. Lane 3 is the column wash with high and low salt column buffer. Lanes 4 and 6 are elutions 1 and 2, respectively, after cleavage with β-ME. Lanes 5 and 7 are the elutions 1 and 2 after running through the polishing chitin column to remove uncleaved MhuD-intein fusion protein.

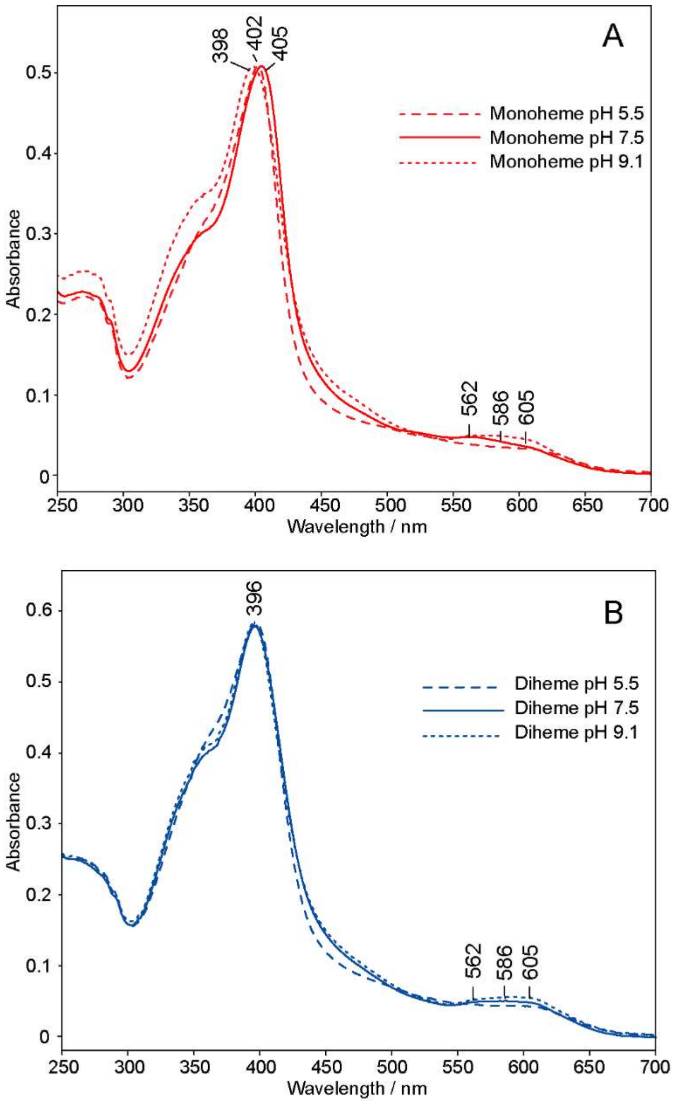

Figure S2. UV-vis spectra of ferric mono- and diheme MhuD under different pH conditions. Spectra were measured at room temperature for (A) monoheme MhuD and (B) diheme MhuD at pH 5.5 (dashed line), pH 7.5 (solid line), and pH 9.1 (dotted line).

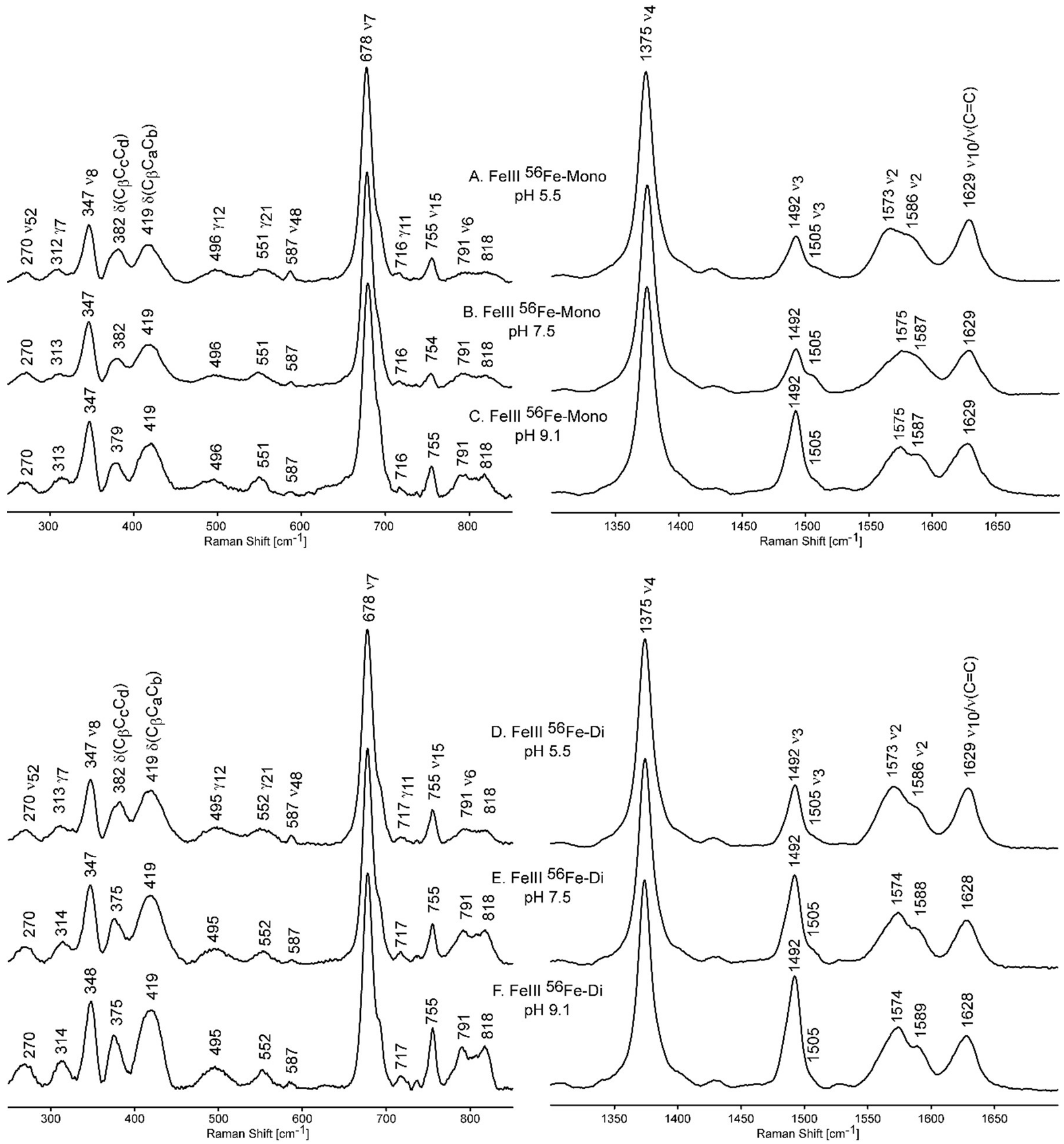

Figure S3. rR spectra of ferric mono- and diheme MhuD under different pH conditions. Shown are spectra in the low frequency (left) and high frequency (right) regions of ferric monoheme MhuD at pH 5.5 (A), 7.5 (B), and 9.1 (C) and ferric diheme MhuD at pH 5.5 (D), 7.5 (E), and 9.1 (F) measured with 406.7 nm excitation line, and 10 mW laser power.

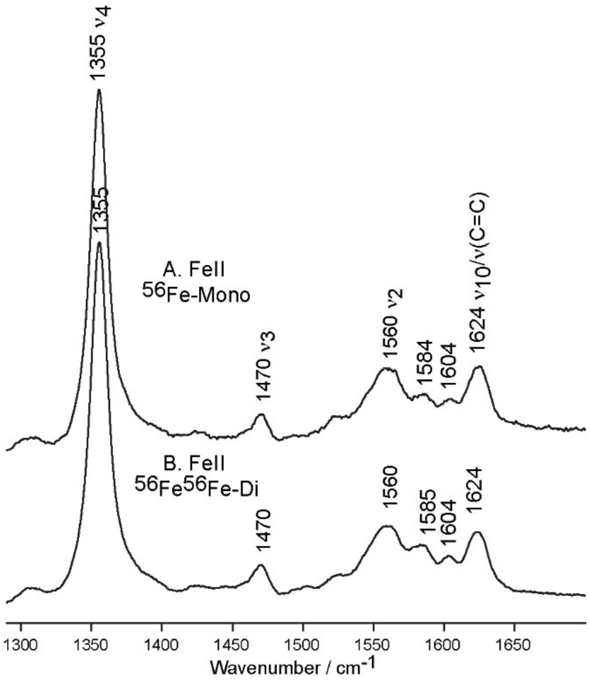

Figure S4. rR spectra in the HF region of ferrous MhuD. Spectra are shown for (A) ferrous ^{56}Fe-monoheme MhuD and (B) ferrous ^{56}Fe^{56}Fe-diheme MhuD in 100 mM potassium phosphate, pH 7.5 measured with 441.6 nm excitation line at laser power of ~10 mW.

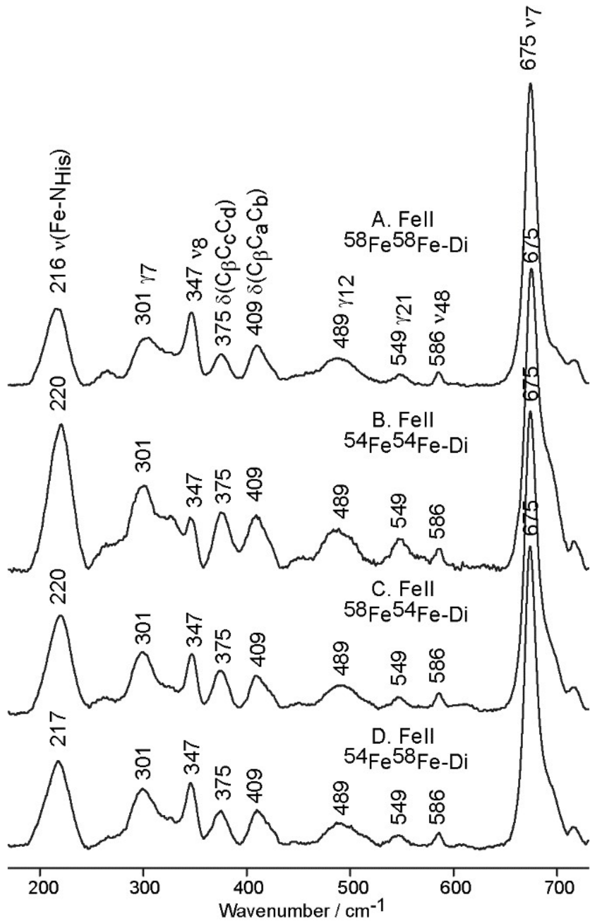

Figure S5. rR spectra in the low frequency region of ferrous diheme MhuD samples with isotopically labeled hemes. Shown are the spectra for ferrous (A) $^{58}Fe^{58}Fe$-diheme, (B) $^{54}Fe^{54}Fe$-diheme, (C) $^{58}Fe^{54}Fe$-diheme, and (D) $^{54}Fe^{58}Fe$-diheme MhuD in 100 mM potassium phosphate, pH 7.5 measured with 441.6 nm excitation line at laser power of ~10 mW.

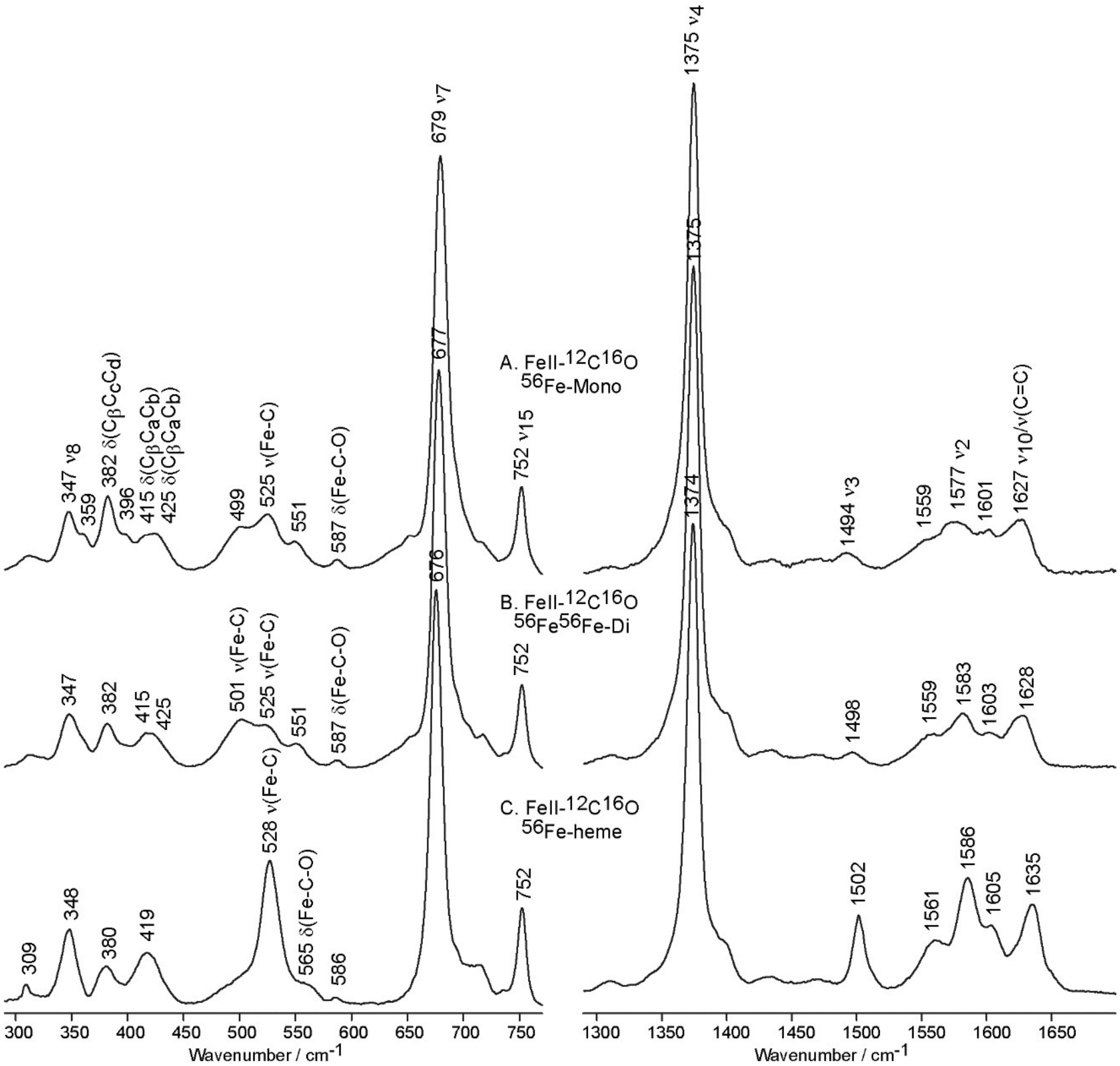

Figure S6. rR spectra of ferrous-$^{12}C^{16}O$ ligated MhuD and free heme. The LF (left) and HF (right) regions are shown for A) ^{56}Fe-monoheme MhuD (A), $^{56}Fe^{56}Fe$-diheme MhuD (B) and free ^{56}Fe-PPIX (C). Samples were measured in 100 mM potassium phosphate, pH 7.5 at room temperature with 413.1 nm excitation line at laser power of 1-2 mW.

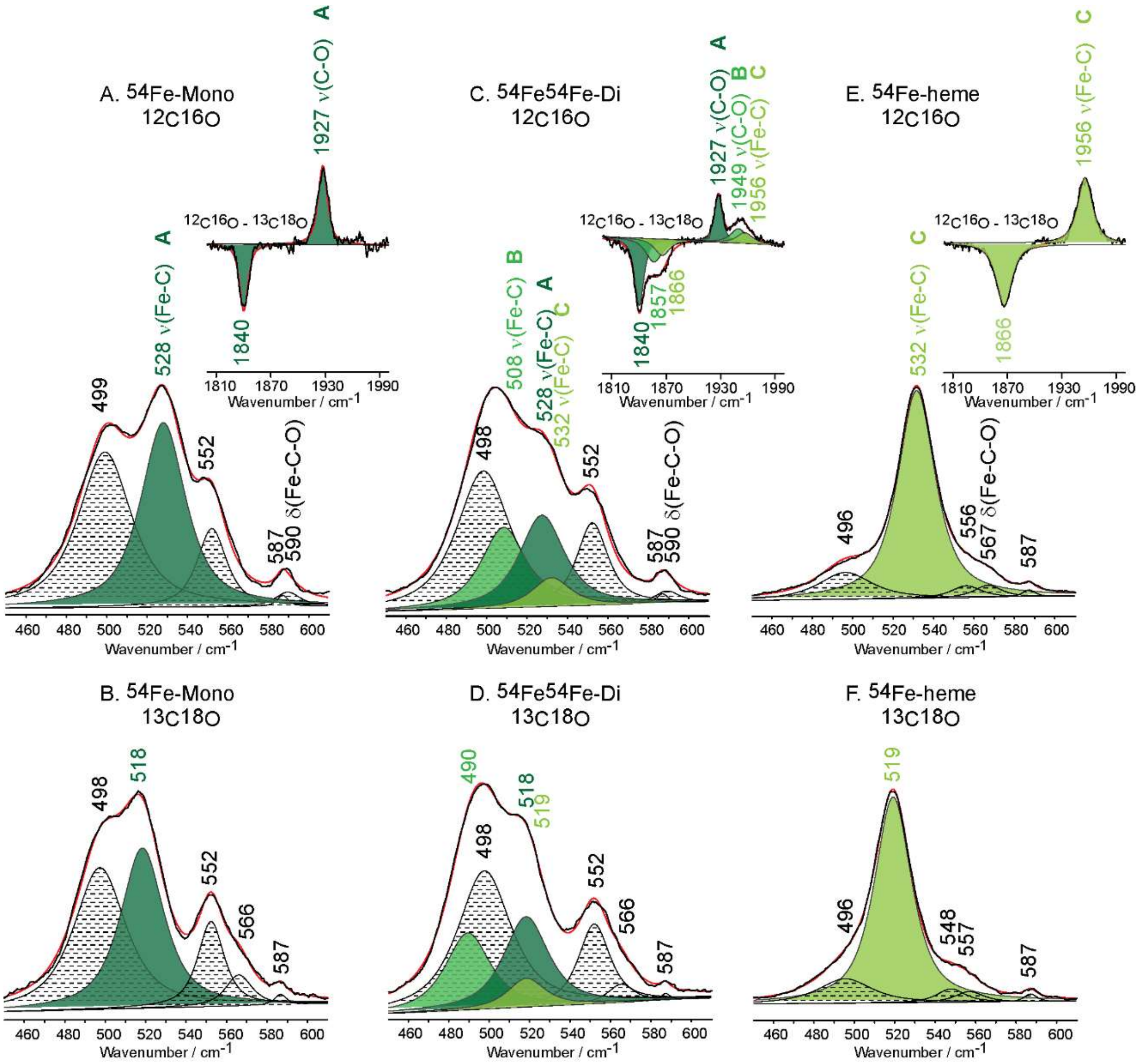

Figure S7. Deconvoluted rR spectra of ferrous-CO adducts of ^{54}Fe MhuD and free heme samples. Shown are ^{54}Fe-monoheme MhuD with $^{12}C^{16}O$ (A) and $^{13}C^{18}O$ (B) isotopes, ^{54}Fe^{54}Fe-diheme MhuD with $^{12}C^{16}O$ (C) and $^{13}C^{18}O$ (D) isotopes, and free ^{54}Fe-PPIX with $^{12}C^{16}O$ (E) and $^{13}C^{18}O$ (F) isotopes. The insets in (A), (C) and (E) show the $^{12}C^{16}O$ - $^{13}C^{18}O$ difference spectrum in the HF region for each respective sample. For each spectrum the black line represents the experimental data, and the red line represents the trace fitted with a mixed 75%/25% Lorentzian/Gaussian function. The black and white fitted peaks with dotted lines represent heme modes and δ(Fe-C-O) modes. The ν(Fe-C) and ν(C-O) modes for CO conformers A, B and C are represented by dark green, medium green and light green peaks, respectively.

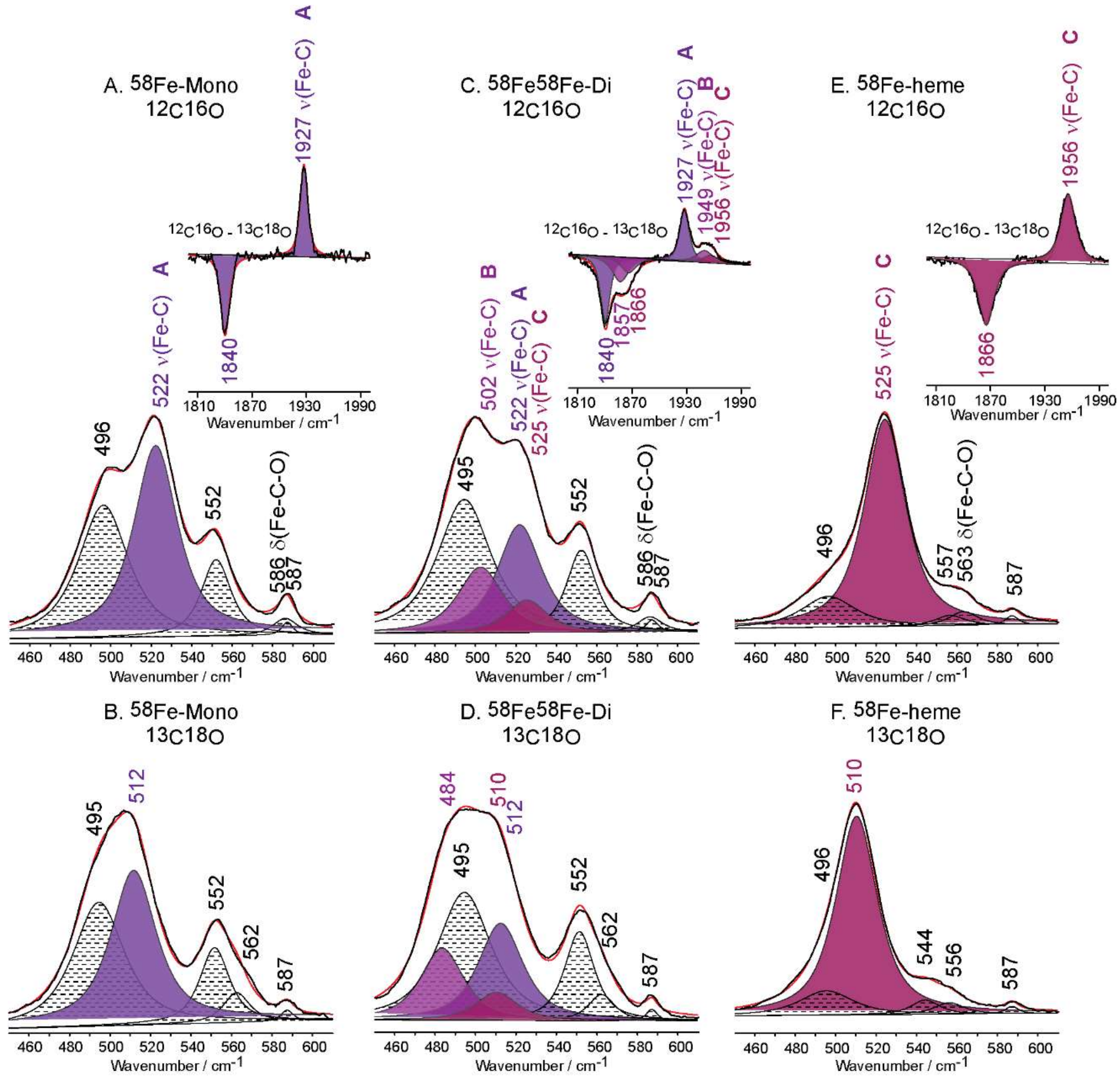

Figure S8. Deconvoluted rR spectra of ferrous-CO adducts of ^{58}Fe MhuD and free heme samples. Shown are ^{58}Fe-monoheme MhuD with ^{12}C^{16}O (A) and ^{13}C^{18}O (B) isotopes, ^{58}Fe^{58}Fe-diheme MhuD with ^{12}C^{16}O (C) and ^{13}C^{18}O (D) isotopes, and free ^{58}Fe-PPIX with ^{12}C^{16}O (E) and ^{13}C^{18}O (F) isotopes. The insets in (A), (C) and (E) show the ^{12}C^{16}O - ^{13}C^{18}O difference spectrum in the HF region for each respective sample. For each spectrum the black line represents the experimental data, and the red line represents the trace fitted with a mixed 75%/25% Lorentzian/Gaussian function. The black and white fitted peaks with dotted lines represent heme modes and δ(Fe-C-O) modes. The ν(Fe-C) and ν(C-O) modes for CO conformers A, B and C are represented by violet, magenta and red peaks, respectively.

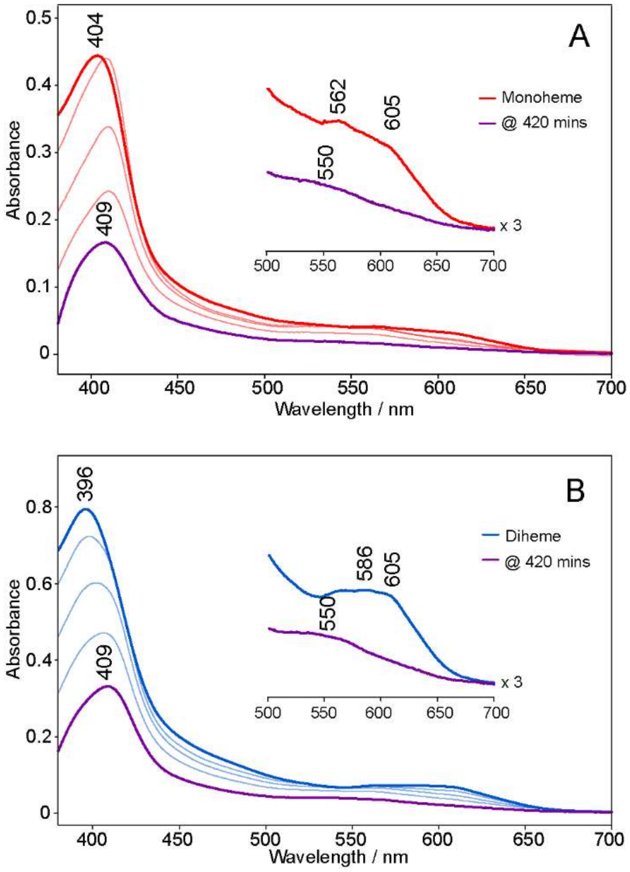

Figure S9. UV-vis spectra of POR activity assays for MhuD. Assays were carried out at room temperature for mono- (A) and diheme (B) MhuD and the reactions contained 5 μM protein concentration of mono- or diheme MhuD in 100 mM KP_i, pH 7.5 with 1250 units/mL catalase, 75 units/mL superoxide dismutase, 10 mM EDTA, 100 μM NADPH and reactions were initiated by addition of 50 nM POR. Spectra were measured intermittently over the course of seven hours.

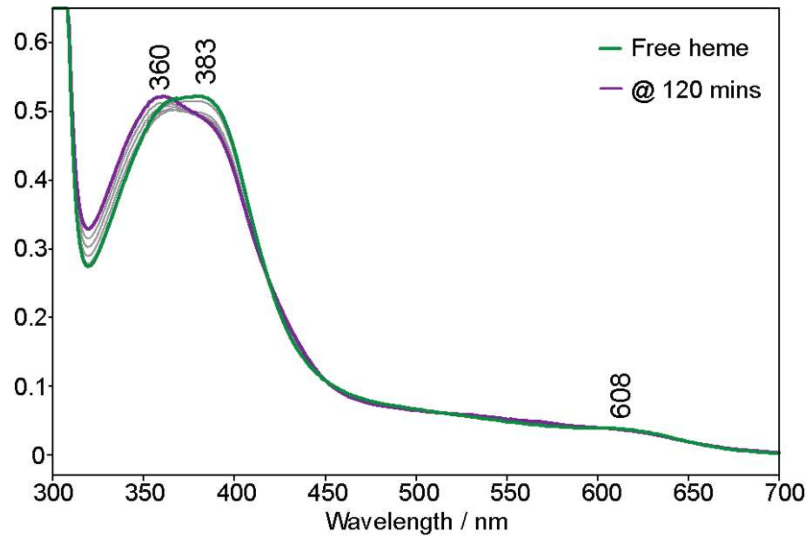

Figure S10. UV-vis spectra of the ascorbate control assay for free heme. The assay was carried out at room temperature and the reaction mixture contained 10 μM hemin-chloride in 100 mM potassium phosphate, pH 7.5 with 1250 units/mL catalase, 75 units/mL superoxide dismutase, 10 mM EDTA, and the reaction was initiated by addition of 10 mM sodium ascorbate. Spectra were measured every 15 minutes for 2 hours. The thick green and purple spectra are 0 and 120 minutes after addition of ascorbate, respectively.

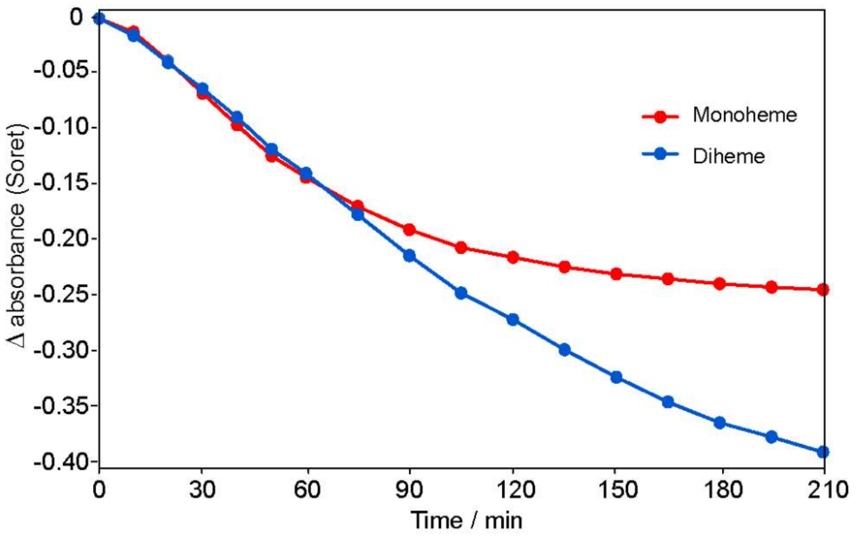

Figure S11. Plot of Δabsorbance of the Soret peak over time for the ascorbate assays. The UV-vis spectral data was obtained from the ascorbate activity assays of monoheme (red) and diheme (blue) MhuD from Figure 9 in the main text.

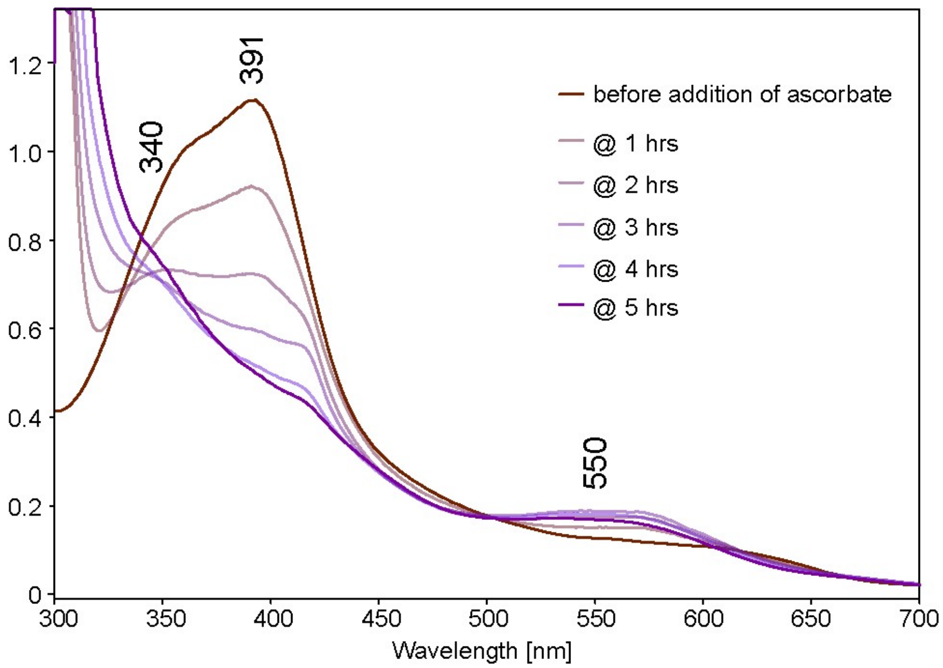

Figure S12. UV-vis spectra of ascorbate assay of MhuD with excess heme. Apo MhuD (4 µM) was incubated with 5-fold excess of hemin chloride (20 µM) for one hour until the absorption spectrum no longer changed to indicate MhuD was saturated with heme in the diheme form. The assay was performed in 50 mM potassium phosphate, pH 6.0 with 1500 units/mL of catalase, 1550 units/mL of superoxide dismutase, 10 mM EDTA, and the reaction was initiated with 10 mM sodium ascorbate. The reaction was monitored over the course of 5 hours, while keeping the sample protected from light at 37°C between spectra. Spectra are shown for the sample before adding ascorbate (brown spectrum) and 1 hour, 2 hours, 3 hours, 4 hours, and 5 hours (dark purple spectrum) after ascorbate was added.

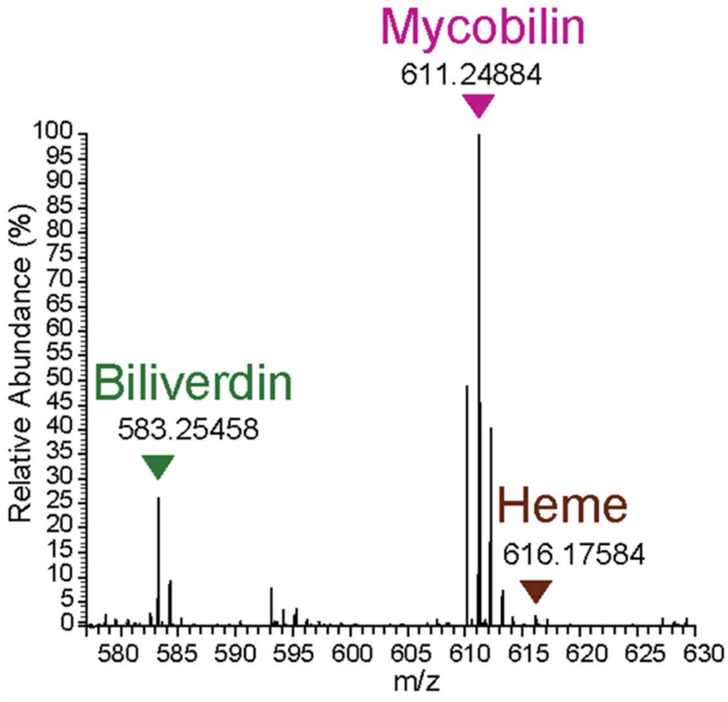

Figure S13. Electrospray ionization mass spectrometry (ESI-MS) spectrum of the crude products of heme degradation by diheme MhuD. The activity assay used ascorbate as an electron donor and was carried out in the presence of catalase and superoxide dismutase at 37 °C. The reaction was allowed to proceed for 140 minutes prior to analysis of the products.

Supplementary tables

Table S1. pH dependence of Soret and Q-band wavelengths (nm) in the UV-vis spectra of mono- and diheme MhuD, IsdG (32), IsdI (32), and rat HO-1 (28, 57) in the ferric state.

	Acidic pH	Neutral pH	Basic pH
Monoheme MhuD	402, 562, 586, 605	405, 562, 586, 605	398, 562, 586, 605
Diheme MhuD	397, 562, 586, 605	396, 562, 586, 605	395, 562, 586, 605
IsdG	410, 540, 563	412[a], 485[a], 520[a], 570[a]	413, 486, 517, 580
IsdI	402, 507, 630	410[a], 485[a], 515[a], 580[a]	413[a], 486[a], 517[a], 580[a]
Rat HO-1	404, 500, 631	406, 500, 539, 575, 632	413, 540, 575

[a] Approximate wavelengths were extrapolated from published spectra when exact wavelengths were not reported/labeled. For IsdI at basic pH, wavelengths were reported to be "very similar to IsdG" (32).

Table S2. Effect of pH on the frequencies of Raman shifts (cm^{-1}) for spin state marker modes ν_2 and ν_3 for mono- and diheme MhuD, IsdG (32), IsdI (32), and rat HO-1 (28) in the ferric state.

	ν_2		ν_3	
	Acidic pH	Alkaline pH	Acidic pH	Alkaline pH
Monoheme MhuD	1573[a], 1586[a]	1574[a], 1588[a]	1492, 1505	1492, 1505
Diheme MhuD	1572[a], 1586[a]	1574[a], 1588[a]	1493, 1505	1492, 1505
IsdG	1560, 1580	1563, 1586	1490, 1506	1484
IsdI	1561	1563	1491	1485
Rat HO-1	1565	1582	1483	1503

[a] Selected frequencies are an approximation due to overlap with other heme modes.

Table S3. Ferrous-CO rR spectral deconvolution data of 1800-2000 cm^{-1} region for free heme samples containing various isotopes of Fe.

	^{54}Fe-PPIX		^{56}Fe-PPIX		^{58}Fe-PPIX	
Peak #	1	2	1	2	1	2
Peak type	$\nu(^{13}C-^{18}O)$	$\nu(^{12}C-^{16}O)$	$\nu(^{13}C-^{18}O)$	$\nu(^{12}C-^{16}O)$	$\nu(^{13}C-^{18}O)$	$\nu(^{12}C-^{16}O)$
Center X (cm^{-1})	1866	1956	1866	1956	1866	1956
Width (cm^{-1})	21	20	20	19	21	20
Peak Area	-2786	2787	-2285	2570	-2828	2698
ΔExp. (cm^{-1})	90		90		90	
ΔTheo. (cm^{-1})	92		92		92	

Table S4. Ferrous-CO rR spectral deconvolution data of 1800-2000 cm^{-1} region for monoheme MhuD samples containing various isotopes of heme Fe.

	^{54}Fe-monoheme		^{56}Fe-monoheme		^{58}Fe-monoheme	
Peak #	1	2	1	2	1	2
Peak type	ν(^{13}C-^{18}O)	ν(^{12}C-^{16}O)	ν(^{13}C-^{18}O)	ν(^{12}C-^{16}O)	ν(^{13}C-^{18}O)	ν(^{12}C-^{16}O)
Center X (cm^{-1})	1840	1927	1840	1927	1840	1927
Width (cm^{-1})	11	12	12	11	11	10
Peak Area	-825	1068	-755	676	-1199	1236
ΔExp. (cm^{-1})	87		87		87	
ΔTheo. (cm^{-1})	90		90		90	

Table S5. Free ^{56}Fe-heme ferrous-CO rR spectral deconvolution data of 450-610 cm^{-1} region.

	$^{12}C^{16}O$					$^{13}C^{16}O$					$^{13}C^{18}O$				
Peak #	1	2	3	4	5	1	2	3	4	5	1	2	3	4	5
Peak type[a]	hm	ν	hm	δ	hm	hm	ν	δ	hm	hm	hm	ν	δ	hm	hm
Center X (cm^{-1})	496	528	556	565	586	496	523	549	557	587	496	513	546	557	586
Width (cm^{-1})	31	21	17	16	7	30	22	15	17	7	31	22	16	17	6
Peak Area	1963	11125	500	646	189	1731	9326	564	432	30	2034	14841	773	696	97
ΔExp. (cm^{-1})							5	16				15	19		
ΔTheo. (cm^{-1})							6					17			

[a] The peak type abbreviations stand for the following: hm is heme mode, ν is ν(Fe-C), and δ is δ(Fe-C-O).

Table S6. ^{56}Fe-monoheme MhuD ferrous-CO rR spectral deconvolution data of 450-610 cm^{-1} region.

	$^{12}C^{16}O$					$^{13}C^{16}O$					$^{13}C^{18}O$				
Peak #	1	2	3	4	5	1	2	3	4	5	1	2	3	4	5
Peak type[a]	hm	ν	hm	hm	δ	hm	ν	hm	δ	hm	hm	ν	hm	δ	hm
Center X (cm^{-1})	497	525	551	587	588	497	521	551	567	587	496	515	551	564	587
Width (cm^{-1})	31	26	17	7	15	31	26	17	15	7	31	26	17	15	7
Peak Area	9487	11000	2827	232	588	12909	14011	4007	1299	235	16302	17531	5907	955	359
ΔExp. (cm^{-1})							4		21			10		24	
ΔTheo. (cm^{-1})							6					17			

[a] The peak type abbreviations stand for the following: hm is heme mode, ν is ν(Fe-C), and δ is δ(Fe-C-O).

Table S7. Ferrous-CO rR spectral deconvolution data of 1800-2000 cm^{-1} region for diheme MhuD samples bound with two of the same isotopes of either ^{54}Fe-, ^{56}Fe- or ^{58}Fe-PPIX.

	$^{54}Fe^{54}Fe$-diheme						$^{56}Fe^{56}Fe$-diheme						$^{58}Fe^{58}Fe$-diheme					
Peak #	1	2	3	4	5	6	1	2	3	4	5	6	1	2	3	4	5	6
Peak type[a]	**νA**	**νB**	**νC**	**νA**	**νB**	**νC**	**νA**	**νB**	**νC**	**νA**	**νB**	**νC**	**νA**	**νB**	**νC**	**νA**	**νB**	**νC**
Center X (cm^{-1})	1840	1857	1866	1927	1949	1956	1840	1857	1866	1927	1949	1956	1840	1857	1866	1927	1949	1956
Width (cm^{-1})	12	21	20	10	20	20	12	21	20	12	21	20	12	21	20	12	21	20
Peak Area	-890	-525	-338	526	301	224	-509	-433	-318	461	254	174	-1206	-766	-440	966	361	215
ΔExp. (cm^{-1})	87	92	90				87	92	90				87	92	90			
ΔTheo. (cm^{-1})	90	92	92				90	92	92				90	92	92			
Ratio ν[b]	1	0.590	0.380	1	0.572	0.426	1	0.851	0.625	1	0.551	0.377	1	0.635	0.365	1	0.374	0.223

[a] The peak type abbreviations νA, νB and νC designate ν(C-O) modes corresponding to their respective CO conformers. Peaks numbered 1-3 and 4-6 indicate samples bound with $^{13}C^{18}O$ and $^{12}C^{16}O$ isotopes, respectively.
[b] The 'Ratio ν' values are the ν(C-O) peak area ratios for each conformer relative to conformer A for a particular isotope of the CO ligand.

Table S8. $^{56}Fe^{56}Fe$-diheme MhuD ferrous-CO rR spectral deconvolution data in the 450-610 cm^{-1} region.

	$^{12}C^{16}O$							$^{13}C^{16}O$							$^{13}C^{18}O$						
Peak #	1	2	3	4	5	6	7	1	2	3	4	5	6	7	1	2	3	4	5	6	7
Peak type[a]	hm	ν**B**	ν**A**	ν**C**	hm	hm	δ	hm	ν**B**	ν**A**	ν**C**	hm	δ	hm	ν**B**	hm	ν**C**	ν**A**	hm	δ	hm
Center X (cm^{-1})	496	505	525	528	551	587	588	496	499	521	523	551	567	587	487	496	513	515	551	564	587
Width (cm^{-1})	31	26	26	22	17	7	15	31	26	26	22	17	15	7	26	31	22	26	17	15	7
Peak Area	6806	3025	4677	1391	2439	154	382	6562	3207	5177	1729	2477	653	134	6360	15287	2852	9812	5873	1542	661
ΔExp. (cm^{-1})									6	4	5		21		18		15	10		24	
ΔTheo. (cm^{-1})									6	6	6				17		17	17			
Ratio ν[b]		0.647	1	0.297					0.619	1	0.334				0.648		0.291	1			

[a] The peak type abbreviations stand for the following: hm is heme mode, ν is ν(Fe-C), and δ is δ(Fe-C-O). The ν(Fe-C) modes are labeled A, B and C corresponding to their respective CO conformers mentioned in the text.
[b] The 'Ratio ν' values are the peak area ratios for ν(Fe-C) of each conformer relative to conformer A for a particular isotope of the CO ligand.

Table S9. Free ^{54}Fe-heme ferrous-CO rR spectral deconvolution data of 450-610 cm^{-1} region.

	$^{12}C^{16}O$					$^{13}C^{18}O$				
Peak #	1	2	3	4	5	1	2	3	4	5
Peak type[a]	hm	ν	hm	δ	hm	hm	ν	δ	hm	hm
Center X (cm^{-1})	496	532	556	567	587	496	519	548	557	587
Width (cm^{-1})	31	22	17	16	7	31	22	16	17	7
Peak Area	2002	11523	528	493	133	2003	12487	621	490	159
ΔExp. (cm^{-1})							13	19		
ΔTheo. (cm^{-1})							17			

[a] The peak type abbreviations stand for the following: hm is heme mode, ν is ν(Fe-C), and δ is δ(Fe-C-O).

Table S10. ^{54}Fe-monoheme MhuD ferrous-CO rR spectral deconvolution data in the 450-610 cm^{-1} region.

	$^{12}C^{16}O$					$^{13}C^{18}O$				
Peak #	1	2	3	4	5	1	2	3	4	5
Peak type[a]	hm	ν	hm	hm	δ	hm	ν	hm	δ	hm
Center X (cm^{-1})	499	528	552	587	590	498	518	552	566	587
Width (cm^{-1})	31	26	17	7	15	31	26	16	15	7
Peak Area	11870	12278	3509	189	509	7965	7810	2602	938	147
ΔExp. (cm^{-1})							10		24	
ΔTheo. (cm^{-1})							17			

[a] The peak type abbreviations stand for the following: hm is heme mode, ν is ν(Fe-C), and δ is δ(Fe-C-O).

Table S11. **$^{54}Fe^{54}Fe$-diheme MhuD ferrous-CO rR spectral deconvolution data in the 450-610 cm^{-1} region.**

	$^{12}C^{16}O$							$^{13}C^{18}O$						
Peak #	1	2	3	4	5	6	7	1	2	3	4	5	6	7
Peak type[a]	hm	ν**B**	ν**A**	ν**C**	hm	hm	δ	ν**B**	hm	ν**A**	ν**C**	hm	δ	hm
Center X (cm^{-1})	498	508	528	532	552	587	590	490	498	518	519	552	566	587
Width (cm^{-1})	31	26	26	22	18	6	15	26	31	26	22	18	15	6
Peak Area	10565	5345	6134	1651	3944	163	434	6108	12966	7357	1972	4666	895	122
ΔExp. (cm^{-1})								18		10	13		24	
ΔTheo. (cm^{-1})								17		17	17			
Ratio ν[b]		0.871	1	0.269				0.830		1	0.268			

[a] The peak type abbreviations stand for the following: hm is heme mode, ν is ν(Fe-C), and δ is δ(Fe-C-O). The ν(Fe-C) modes are labeled A, B and C corresponding to their respective CO conformers mentioned in the text.
[b] The 'Ratio ν' values are the peak area ratios for ν(Fe-C) of each conformer relative to conformer A for a particular isotope of the CO ligand.

Table S12. Free ^{58}Fe-heme ferrous-CO rR spectral deconvolution data of 450-610 cm^{-1} region.

	$^{12}C^{16}O$					$^{13}C^{18}O$				
Peak #	1	2	3	4	5	1	2	3	4	5
Peak type[a]	hm	ν	hm	δ	hm	hm	ν	δ	hm	hm
Center X (cm^{-1})	496	525	557	563	587	496	510	544	556	587
Width (cm^{-1})	31	24	17	16	7	31	24	16	17	7
Peak Area	3169	17370	613	788	235	2654	17608	880	738	206
ΔExp. (cm^{-1})							15	19		
ΔTheo. (cm^{-1})							17			

[a] The peak type abbreviations stand for the following: hm is heme mode, ν is ν(Fe-C), and δ is δ(Fe-C-O).

Table S13. ^{58}Fe-monoheme MhuD ferrous-CO rR spectral deconvolution data in the 450-610 cm^{-1} region.

	$^{12}C^{16}O$					$^{13}C^{18}O$				
Peak #	1	2	3	4	5	1	2	3	4	5
Peak type[a]	hm	ν	hm	δ	hm	hm	ν	hm	δ	hm
Center X (cm^{-1})	496	522	552	586	587	495	512	552	562	587
Width (cm^{-1})	30	26	17	15	7	30	26	17	15	7
Peak Area	14733	18845	5343	935	342	9771	10815	3652	1283	224
ΔExp. (cm^{-1})							10		24	
ΔTheo. (cm^{-1})							17			

[a] The peak type abbreviations stand for the following: hm is heme mode, ν is ν(Fe-C), and δ is δ(Fe-C-O).

Table S14. $^{58}Fe^{58}Fe$-diheme MhuD ferrous-CO rR spectral deconvolution data in the 450-610 cm^{-1} region.

	$^{12}C^{16}O$							$^{13}C^{18}O$						
Peak #	1	2	3	4	5	6	7	1	2	3	4	5	6	7
Peak type[a]	hm	ν**B**	ν**A**	ν**C**	hm	δ	hm	ν**B**	hm	ν**C**	ν**A**	hm	δ	hm
Center X (cm^{-1})	495	502	522	525	552	586	587	484	495	510	512	552	562	587
Width (cm^{-1})	31	26	26	24	17	15	7	26	31	24	26	17	15	6
Peak Area	12947	5535	9089	2511	4753	704	285	6957	15208	2725	10100	6313	1654	318
ΔExp. (cm^{-1})								18		15	10		24	
ΔTheo. (cm^{-1})								17		17	17			
Ratio ν[b]		0.609	1	0.276				0.689		0.270	1			

[a] The peak type abbreviations stand for the following: hm is heme mode, ν is ν(Fe-C), and δ is δ(Fe-C-O). The ν(Fe-C) modes are labeled A, B and C corresponding to their respective CO conformers mentioned in the text.
[b] The 'Ratio ν' values are the peak area ratios for ν(Fe-C) of each conformer relative to conformer A for a particular isotope of the CO ligand.

Table S15. $^{54}Fe^{58}Fe$-diheme MhuD ferrous-CO rR spectral deconvolution data in the 450-610 cm^{-1} region.

	$^{12}C^{16}O$							$^{13}C^{18}O$						
Peak #	1	2	3	4	5	6	7	1	2	3	4	5	6	7
Peak type[a]	hm	ν**B**	ν**A**	ν**C**	hm	δ	hm	ν**B**	hm	ν**A**	ν**C**	hm	δ	hm
Center X (cm^{-1})	495	502	522	532	552	586	587	484	495	512	519	552	562	587
Width (cm^{-1})	31	26	26	22	17	15	6	26	31	26	22	17	15	7
Peak Area	10098	4875	7970	2184	3598	371	106	4514	10470	7299	2112	4330	1054	319
ΔExp. (cm^{-1})								18		10	13		24	
ΔTheo. (cm^{-1})								17		17	17			
Ratio ν[b]		0.612	1	0.274				0.618		1	0.289			

[a] The peak type abbreviations stand for the following: hm is heme mode, ν is ν(Fe-C), and δ is δ(Fe-C-O). The ν(Fe-C) modes are labeled A, B and C corresponding to their respective CO conformers mentioned in the text.
[b] The 'Ratio ν' values are the peak area ratios for ν(Fe-C) of each conformer relative to conformer A for a particular isotope of the CO ligand.

Table S16. $^{58}Fe^{54}Fe$-diheme MhuD ferrous-CO rR spectral deconvolution data in the 450-610 cm^{-1} region.

	$^{12}C^{16}O$							$^{13}C^{18}O$						
Peak #	1	2	3	4	5	6	7	1	2	3	4	5	6	7
Peak type[a]	hm	ν**B**	ν**C**	ν**A**	hm	hm	δ	ν**B**	hm	ν**C**	ν**A**	hm	δ	hm
Center X (cm^{-1})	498	508	525	528	552	587	590	490	498	510	518	552	566	587
Width (cm^{-1})	31	26	24	26	17	6	15	26	31	24	26	17	15	6
Peak Area	10410	5213	2313	7332	4067	157	434	5119	10558	1977	7681	4235	927	166
ΔExp. (cm^{-1})								18		15	10		24	
ΔTheo. (cm^{-1})								17		17	17			
Ratio ν[b]		0.711	0.315	1				0.666		0.257	1			

[a] The peak type abbreviations stand for the following: hm is heme mode, ν is ν(Fe-C), and δ is δ(Fe-C-O). The ν(Fe-C) modes are labeled A, B and C corresponding to their respective CO conformers mentioned in the text.
[b] The 'Ratio ν' values are the peak area ratios for ν(Fe-C) of each conformer relative to conformer A for a particular isotope of the CO ligand.

Table S17. Ferrous-CO rR spectral deconvolution data of 1800-2000 cm^{-1} region for diheme MhuD samples bound with one of each of the isotopically labeled hemes, ^{54}Fe- and ^{58}Fe-PPIX.

	$^{54}Fe^{58}Fe$-diheme						$^{58}Fe^{54}Fe$-diheme					
Peak #	1	2	3	4	5	6	1	2	3	4	5	6
Peak type[a]	**νA**	**νB**	**νC**	**νA**	**νB**	**νC**	**νA**	**νB**	**νC**	**νA**	**νB**	**νC**
Center X (cm^{-1})	1840	1857	1866	1927	1949	1956	1840	1857	1866	1927	1949	1956
Width (cm^{-1})	12	21	20	12	20	20	12	21	20	12	20	20
Peak Area	-661	-339	-215	672	275	152	-821	-608	-387	830	322	167
ΔExp. (cm^{-1})	87	92	90				87	92	90			
ΔTheo. (cm^{-1})	90	92	92				90	92	92			
Ratio ν[b]	1	0.513	0.325	1	0.409	0.226	1	0.741	0.471	1	0.388	0.201

[a] The peak type abbreviations νA, νB and νC designate ν(C-O) modes corresponding to their respective CO conformers. Peaks numbered 1-3 and 4-6 indicate samples bound with $^{13}C^{18}O$ and $^{12}C^{16}O$ isotopes, respectively.
[b] The 'Ratio ν' values are the ν(C-O) peak area ratios for each conformer relative to conformer A for a particular isotope of the CO ligand.
